# Synthetic and computational assessment of a chiral metal–organic framework catalyst for predictive asymmetric transformation†

**DOI:** 10.1039/d0sc03364b

**Published:** 2020-08-06

**Authors:** Jérôme Canivet, Elise Bernoud, Jonathan Bonnefoy, Alexandre Legrand, Tanya K. Todorova, Elsje Alessandra Quadrelli, Caroline Mellot-Draznieks

**Affiliations:** Univ. Lyon, Université Claude Bernard Lyon 1, CNRS, IRCELYON UMR 5256 2 Avenue Albert Einstein 69626 Villeurbanne France jerome.canivet@ircelyon.univ-lyon1.fr; Laboratoire de Chimie des Processus Biologiques, Collège de France, Sorbonne Université, CNRS UMR 8229, PSL Research University 11 Place Marcelin Berthelot Paris 75231 Cedex 05 France caroline.mellot-draznieks@college-de-france.fr; Univ. Lyon, Université Claude Bernard Lyon 1, CNRS, C2P2 UMR 5265 43 Boulevard du 11 Novembre 1918 69616 Villeurbanne France

## Abstract

Understanding and controlling molecular recognition mechanisms at a chiral solid interface is a continuously addressed challenge in heterogeneous catalysis. Here, the molecular recognition of a chiral peptide-functionalized metal–organic framework (MOF) catalyst towards a pro-chiral substrate is evaluated experimentally and *in silico*. The MIL-101 metal–organic framework is used as a macroligand for hosting a Noyori-type chiral ruthenium molecular catalyst, namely (benzene)Ru@MIL-101-NH-Gly-Pro. Its catalytic perfomance toward the asymmetric transfer hydrogenation (ATH) of acetophenone into *R*- and *S*-phenylethanol are assessed. The excellent match between the experimentally obtained enantiomeric excesses and the computational outcomes provides a robust atomic-level rationale for the observed product selectivities. The unprecedented role of the MOF in confining the molecular Ru-catalyst and in determining the access of the prochiral substrate to the active site is revealed in terms of highly face-specific host–guest interactions. The predicted surface-specific face differentiation of the prochiral substrate is experimentally corroborated since a three-fold increase in enantiomeric excess is obtained with the heterogeneous MOF-based catalyst when compared to its homogeneous molecular counterpart.

## Abstract 2

Understanding and controlling molecular recognition mechanisms at a chiral solid interface has been addressed in metal–organic framework catalysts for the asymmetric transfer hydrogenation reaction.

## Graphical abstract

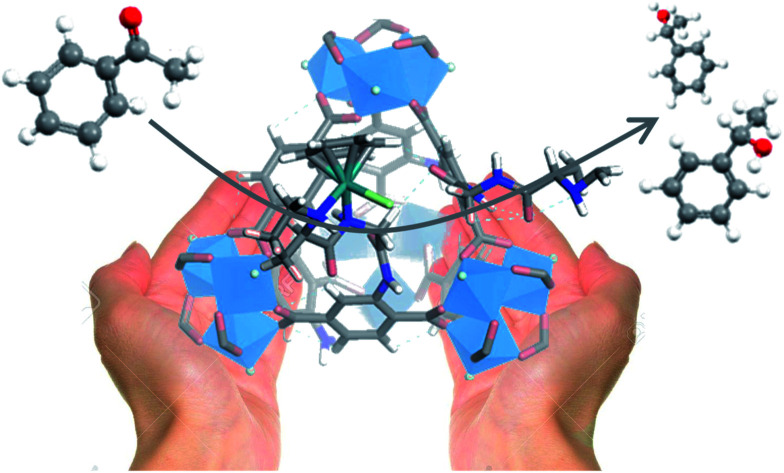

## Introduction

Among marketed drugs, 56% contain a chiral center.^1^ Asymmetric catalysis plays a key role in lowering the environmental impact of the synthetic routes of pure chiral molecules for pharmaceuticals, agrochemicals and flavors,^2^ allowing the elimination, for example, of environmentally costly separation steps between the desired and unwanted enantiomers or between different diastereoisomers. These asymmetric catalytic routes are mostly performed under homogeneous conditions, which still impose potentially costly constraints in terms of isolation of the targeted molecule.

Heterogeneous asymmetric catalysis would in principle overcome this issue, further improving the sustainability of the synthetic process. However, despite numerous interesting approaches and isolated achievements, heterogeneous asymmetric catalysis has not yet reached comparable deployment.^3,4^ There is thus a pressing need to develop new strategies to understand and rationalize asymmetric interactions at the solid's interface with the aim of designing performing heterogeneous chiral catalysts.

The last decade has seen unprecedented progresses in first-principle methods such as density functional theory (DFT) to understand the behavior and promote the rational design of inorganic solid catalysts mainly for bulk chemistry.^5–8^ In this context, crystalline porous materials like Metal–Organic Frameworks (MOF) have been explored as ideal model crystalline structures for *in silico* investigations of catalytic transformations, however challenged by the increasing sophistication of their hybrid organic–inorganic structures.^9–13^

Nevertheless, turning to enantioselective reactions, the computationally-driven rationalization of their catalytic activity in heterogeneous asymmetric transformations is challenging. To the best of our knowledge, such rationalization has never been achieved so far notwithstanding the major analogous achievements in computational molecular catalysis,^14,15^ at least partly because of the complexity of the chemical reactions at the solid's interface when chiral supramolecular interactions come into play.

Here we report the first combined experimental and computational study on chiral porous functionalized hybrids that successfully provides insights into the enantioselective process within novel MOF-based chiral solids and their evaluation for asymmetric catalysis outperforming their homogeneous counterparts. Chiral arene ruthenium prolinamide catalysts are embedded into a MOF host for the first time, and evaluated experimentally in asymmetric transfer hydrogenation (ATH) reaction. To understand the origin of the preferential enantiomer formation within the MOF which outperform the homogeneous counterpart, we investigate computationally the conformations of MOF-confined chiral arene ruthenium prolinamide organometallic complexes. The calculations unveil the crucial role of the surrounding MOF in the supramolecular interactions at the origin of the selectivity in the Ru-catalyzed ATH of acetophenone, selected as a prochiral substrate. On the basis of the *in silico* findings, computationally predicted enantioselectivities in ATH reaction are confronted to the experimental ones and discussed.

## Results and discussion

### MIL-101 as MOF scaffold

**Fig. 1:**
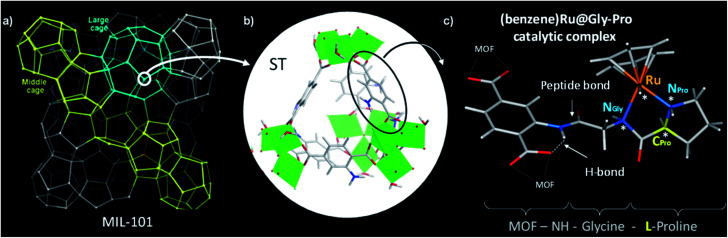
MIL-101 metal–organic framework as macroligand for hosting the chiral ruthenium molecular catalyst, namely (benzene)Ru@MIL-101-NH-Gly-Pro. (a) Schematic representation of the Al-MIL-101-NH_2_ hybrid framework. The hybrid supertetrahedra (ST) are represented as 4-connected corner-sharing nodes (circled in white); (b) detailed view of the hybrid ST showing bdc-NH_2_ linkers (sticks) on edge-positions and the inorganic Al-octahedra trimers (green polyhedra) in corner-position; (c) functionalization of MIL-101's organic linker with a –Gly–Pro dipeptide (here, l-proline) in complex with (benzene)Ru. The asymmetric atoms (N_Pro_, C_Pro_, N_Gly_ and Ru) of the catalytic graft are noted with asterisks. Color code: C, H (grey), N (blue), O (red), Al (green), Ru (orange).

We selected the extra-large pore aminated framework Al-MIL-101-NH_2_,^16^ which is isostructural to Cr-MIL-101 (ref. 17,18) and benefits from a mesopore ideally sized to host large grafted species such as catalytic complexes while preserving their accessibility to substrates (Fig. 1a).^19^ Al-MIL-101-NH_2_ is made of octahedral trimeric aluminum(iii) clusters linked by 2-aminoterephthalate linkers (bdc-NH_2_) assembled into supertetrahedra building blocks (Fig. 1b). Aiming at synthesizing a novel class of chiral porous solids, we have previously designed a family of peptide-functionalized MOF capable of performing asymmetric transformations such as asymmetric aldol catalysis.^20,21^ Atomic-level insights into such peptide-functionalized solids were gained from DFT calculations and ^15^N NMR of the grafted peptides within the pores, typified in the MIL-68-NH-Gly-Pro solid (Gly: glycine, Pro: proline).^22^ With the Gly–Pro graft, the glycine spacer was found to be essential in placing the Pro residue towards the center of the cavity rather than folded towards the MOF hydroxyl groups.

Since molecular transition metal complexes with prolinamide (R–NH–Pro) ligands have already been found to catalyse the asymmetric transfer hydrogenation of ketones,^23–25^ we postulated that their grafting and confinement into the MOF cavity may leverage their enantioselectivity when compared to that of the homogeneous counterparts, in a similar fashion to that reported in mesoporous silica.^26^

Despite numerous examples of homochiral amido-functionalized MOF including prolinamide moieties,^27^ there is no report so far of amido-functionalized MOF used either as a porous macroligand, *i.e.* a solid acting as the organic ligand in the corresponding molecular complex,^28–30^ for such organometallic complexes or for asymmetric transfer hydrogenation catalysis.

Thus we use here the enantiopure Al-MIL-101-NH-Gly-Pro solid,^21^ synthesized with either d- or l-proline, as a porous macroligand for hosting a chiral ruthenium complex (Fig. 1c), noted here (benzene)Ru@MIL-101-NH-Gly-Pro, able to catalyse asymmetric transfer hydrogenation (ATH) reaction (see Section 2 of ESI†).

### Synthesis of MOF-based chiral catalysts

**Fig. 2:**
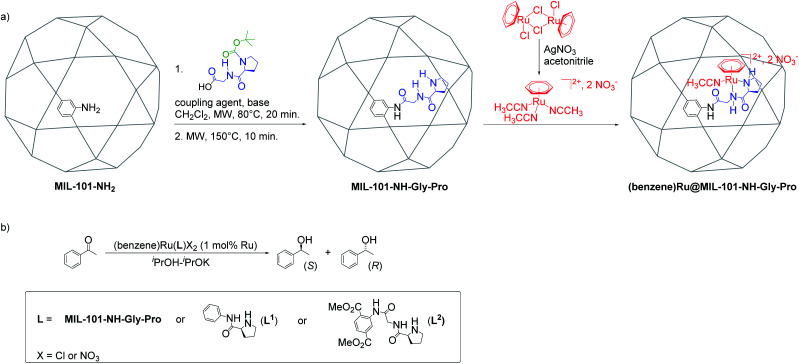
Synthesis and catalytic application of (benzene)Ru@MIL-101-NH-Gly-Pro. (a) Post-synthetic peptide grafting and metalation of the Al-MIL-101-NH_2_ with the chiral benzene ruthenium prolinamide complex; (b) acetophenone asymmetric transfer hydrogenation catalyzed by benzene ruthenium species combined with MOF macroligand or molecular ligands, L^1^ and L^2^ (see Section 4 in ESI†).

The Al-MIL-101-NH-Gly-(l)Pro was prepared from Al-MIL-101-NH_2_ and l-GlyProBoc following our reported two-steps microwave (MW)-assisted protocol whereby 35% of bdc-NH_2_ linkers of the MOF were functionalized with –Gly–Pro dipeptides (Fig. 2a and S10†).^21^ For the post-synthetic metalation of the peptide-functionalized Al-MIL-101-NH-Gly-Pro macroligand, we used a commercially available dimeric (arene)Ru complexes. The procedure first consisted in splitting the dimers into two identical solvated arene ruthenium complexes, whereby the benzene ruthenium dichloride dimer was put in reaction with silver nitrate in acetonitrile (acn) to yield the monomeric dicationic complex, [(C_6_H_6_)Ru(acn)_3_]^2+^ (Fig. 2a). This solvation is accelerated by the irreversible removal of chloro ligands and their precipitation as non-soluble silver chloride.^31,32^ Finally, Al-MIL-101-NH-Gly-(l)Pro reacted with [(C_6_H_6_)Ru(acn)_3_][NO_3_]_2_ to give the (benzene)Ru@MIL-101-NH-Gly-(l)Pro MOF material of interest (Fig. 2a). The (benzene)Ru@MIL-101-NH-Gly-(d)Pro solid was prepared following the same procedure but starting from the Al-MIL-101-NH-Gly-(d)Pro, the latter showing similar physicochemical features to its (l)Pro-containing counterpart.^21^

The powder X-ray diffraction pattern of the (benzene)Ru@MIL-101-NH-Gly-Pro solids corresponds to that of the parent MIL-101 solid, assessing that the crystallinity is preserved upon the successive grafting and metalation steps (Fig. S11†). Brunauer–Emmett–Teller (BET) surface area calculated from the nitrogen adsorption/desorption measurements reveals that the porosity is maintained with a BET surface area of 581 m^2^ g^−1^ and an accessible porous volume of 0.3717 cm^3^ g^−1^ for (benzene)Ru@MIL-101-NH-Gly-(l)Pro (Fig. S12 and S13†).

ICP-OES elemental analysis shows a metal content of 6.7 wt% for Al and 3.9 wt% for Ru. The liquid state ^1^H NMR of dissolved (benzene)Ru@MIL-101-NH-Gly-(l)Pro in HF–H_2_O/DMSO d^6^ evidenced the characteristic peaks of (C_6_H_6_)Ru species at *δ* = 5.7–6.0 ppm as well as a (bdc-NH_2_) : (bdc–NH–Gly–Pro) : [(C_6_H_6_)Ru] ratio of 10 : 4.7 : 3 (Fig. S14†). Altogether, the functionalization yield obtained from ^1^H NMR analysis is in line with the Ru content obtained from ICP-OES analysis, allowing us to propose the formula Al_3_OCl(bdc-NH_2_)_2_(bdc–NH–Gly–(l)Pro)_0.6_[bdc–NH–Gly(l)Pro (C_6_H_6_)Ru(acn) (NO_3_)_2_]_0.4_·4 isopropanol.

It is worth noting that the Ru-metalation was unsuccessful following this procedure using Al-MIL-101-NH-Pro^21^ instead of Al-MIL-101-NH-Gly-Pro as the starting functionalized platform. This can be explained based on the steric hindrance occurring in Al-MIL-101-NH-Pro, whereby the proline's chelating nitrogen is closer to the MOF's wall than in Al-MIL-101-NH-Gly-Pro, which further hinders the access of the arene ruthenium complex. Importantly, the glycine residue hence plays the role of a necessary spacer by placing the proline residue away from the MOF's wall^22^ and allowing its subsequent coordination to the arene ruthenium moiety.

### Experimental evaluation of homogeneous and heterogeneous chiral Ru-prolinamide catalysts in ketone ATH reaction

The catalytic activity of the MOF-supported chiral benzene ruthenium complexes, (benzene)Ru@MIL-101-NH-Gly-Pro, was evaluated for the ATH of acetophenone into phenylethanol in isopropanol using chiral HPLC analysis (Table 1, Fig. S19 and S20†). Both heterogeneous catalysts, with exclusively (l)Pro or (d)Pro, were tested separately. For comparison purposes, two molecular (C_6_H_6_)Ru(L)Cl_2_ analogues were also prepared (Fig. 2b, S16–S18 and Section 4 in ESI†) and assayed (Fig. S21 and S22†), using *N*-phenyl-(l)-prolinamide (L^1^) or (l)-dimethyl-2-(2-(pyrrolidine-2-carboxamido)acetamido)terephthalate (L^2^) as Ru ligands. (benzene)Ru(L^2^) represents the closest molecular analogue of the heterogeneous MOF-supported (benzene)Ru(Gly-(l)Pro) catalyst where carboxylate groups mimic those present in the MOF scaffold.

**Table 1:** Asymmetric transfer hydrogenation of acetophenone.^a^ Comparison of ATH catalytic activity and selectivity of (benzene)Ru@MIL-101-NH-Gly-Pro and molecular Ru complexes with L^1^ and L^2^ ligands

Entry	Catalyst	Temperature (°C)	Yield^b^ (%)	E.e.^b^ (%)
1	(benzene)Ru@MIL-101-NH-Gly-(l)Pro	20	15	38 (*S*)
2	(benzene)Ru@MIL-101-NH-Gly-(l)Pro	40	32	28 (*S*)
3	(benzene)Ru@MIL-101-NH-Gly-(l)Pro	60	90	20 (*S*)
4	(benzene)Ru@MIL-101-NH-Gly-(d)Pro	20	12	21 (*R*)
5	(benzene)Ru(L^1^)Cl_2_^c^	20	85	12 (*R*)
6	(benzene)Ru(L^2^)Cl_2_^c^	20	21	7 (*R*)

a Conditions: 3.9 μmol of Ru (corresponding to 10 mg of MOF catalyst), 2 mg of KOH (36 μmol) in 3 mL of anhydrous isopropanol and 386 mmol of acetophenone (45 μL) at desired temperature for 24 hours.

b Determined by HPLC analysis (AS-H column, hexane : isopropanol = 97 : 3, 0.9 mL min^−1^, 215 nm). The configuration of the major product is given in brackets.

c The catalyst is obtained by mixing *in situ* [(C_6_H_6_)RuCl_2_]_2_ and 1 eq./Ru of the desired l-Pro derived ligand in isopropanol at room temp *prior* to catalytic test.

At 20 °C, the (benzene)Ru@MIL-101-NH-Gly-Pro heterogeneous catalysts lead to enantiomeric excesses (e.e.) of 38% when using (l)Pro and 21% when using (d)Pro (yields = 12–15%, Table 1, entries 1 and 4). The heterogeneous nature of the MOF-based catalytic process was confirmed by split test. After solid catalyst filtration from the supernatant under argon, the solution was allowed to react for further 24 h and no evolution of conversion or enantiomeric excess was observed over time (Fig. S23†). Moreover, the recyclability of the (benzene)Ru@MIL-101-NH-Gly-(l)Pro catalyst was evaluated through 3 consecutive runs by carefully washing the solid with anhydrous isopropanol, whereby no loss of either activity or selectivity was observed (Fig. S24†). The e.e. observed with the MOF-based heterogeneous catalyst are significantly higher than those observed under homogeneous conditions with the molecular analogues of the catalytically active species in the MOF solid.

Chiral Ru-prolinamide molecular species have been reported to show enantiomeric excess in the ATH reaction ranging from 4 to 13% in the reduction of acetophenone when using *N*-phenyl-prolinamide as the Ru ligand.^33^ The e.e. values obtained with the two molecular (benzene)Ru(L^1^) and (benzene)Ru(L^2^) catalysts, while being in a similar range (7–12%) with those reported for the *N*-phenyl-prolinamide Ru complex (4–13%),^33^ are significantly lower than those achieved with the MOF-supported catalysts (21–38%). Regarding the yields, (benzene)Ru(L^2^), the closest structural molecular analogue of the MOF macroligand (see Fig. 2b), shows a yield (21%, Table 1, entry 6) to be compared with that obtained with the (benzene)Ru@MIL-101-NH-Gly-(l)Pro catalyst (15%, Table 1, entry 1). In contrast, (benzene)Ru(L^1^) leads to higher yield (85% yield, Table 1, entry 5).

The difference in yield between (benzene)Ru(L^2^) and (benzene)Ru(L^1^) shows the capacity of substituents on the ancillary ligand to affect the catalytic activity at the ruthenium center. The electron withdrawing group in L^2^ induces a lower electron density at the ruthenium center in (benzene)Ru(L^2^) resulting in its lower activity when compared to that of (benzene)Ru(L^1^). The similarity in yield between (benzene)Ru(L^2^) and the MOF-based catalyst suggests that there is no substantial limitation due to the diffusion of reactant and products within the porous solid.

Regarding the effect of the temperature on the catalytic performances, we observed that increasing the reaction temperature using the MOF-based catalyst favors the reactivity of the ruthenium center at the expense of its selectivity. Upon heating from 20 to 60 °C, the yield increases from 15 to 90% while the e.e. decreases from 38 to 20%, remaining still higher than those observed with the homogeneous catalysts at room temperature.

Overall, the selectivity obtained with the heterogeneous (benzene)Ru@MIL-101-NH-Gly-(l)Pro catalyst is three times higher than that found in analogous homogeneous systems. These experimental results highlight the key role of the MOF macroligand in enhancing the enantioselectivity in the ATH reaction.

When using the (l)Pro-based (benzene)Ru@MIL-101-NH-Gly-(l)Pro catalyst, the main product is the *S*-phenylethanol (entry 1). We also assayed the (d)Pro-based catalyst and similar conversion (12%) and e.e. (21%) were found with the (benzene)Ru@MIL-101-NH-Gly-(d)Pro (Table 1, entry 4) as with the (l)Pro-based one (Table 1, entry 1), detecting the *R*-phenylethanol as the main product, as expected. Remarkably, the inversion of selectivity on the basis of the inversion of the catalytic centre's configuration, which is well-known in molecular catalysts,^34^ is shown to be indeed at play in the solid state, *i.e.* when the Ru-catalyst is hosted in the MOF macroligand. Furthermore, the correlation between the configuration of the proline (with its *R* or *S* carbon) and that of the product (*S* or *R*, respectively) is fully confirmed by these experiments.

Notably, an inversion of the 2-phenylethanol product's configuration is observed between the heterogeneous (Table 1, entries 1–3) and homogeneous (Table 1, entries 5 and 6) (l)Pro-based catalysts, whereby *S* and *R* enantiomers are formed as main products respectively. A reversed enantioselectivity in MOF-supported catalysts compared to homogeneous analogues was also reported for proline-catalyzed asymmetric aldol reaction in two recent studies, both postulating a change in the substrate's activation pathway influenced by either the inorganic MOF node or adsorbed solvent molecules.^35,36^

It thus clearly appears that the MOF scaffold surrounding the Ru catalytic centre is at the origin of the higher enantioselectivity, as well as the enantiomer inversion. To get molecular level insight into the enantioselective mechanism at play within the (benzene)Ru@MIL-101-NH-Gly-Pro catalyst, we implemented a comprehensive DFT-level computational study.

### Computational generation of chiral models

We started with a stepwise computational strategy to find the most likely anchoring positions for the (benzene)Ru(Gly–Pro) catalytic graft into the MOF's cavity. It consisted in the construction of a library of initial models of the (benzene)Ru@MIL-101-NH-Gly-(l)Pro solid, followed by molecular dynamics (MD) simulations and density functional theory (DFT) calculations (see ESI, Section 1.1†). This initial set of periodic constructs was built by covalently grafting the (benzene)Ru(Gly–Pro) moiety through a peptide bond between the amino group of a MOF's organic linker and the proline's carboxylate end, at various positions into the unit-cell of Al-MIL-101-NH_2_. In view of the complexity of this structure, the primitive cell of Al-MIL-101-NH_2_'s crystal structure was used and the grafting was performed within a 6-membered ring of a large cage (Fig. S1†), thus allowing an optimal access for the substrate.

The (benzene)Ru@MIL-101-NH-Gly-(l)Pro periodic constructs were used as starting models to perform 5 ns MD calculations at 298 K. A selection of finite-size models (*ca.* 680 atoms) were then extracted from the MD trajectories of the periodic models and optimized and compared at the dispersion-corrected DFT-D3 level to assess the interactions of the (benzene)Ru(Gly–Pro) graft with the MOF host (Fig. S2 and Section 1.4 in ESI†).

**Fig. 3:**
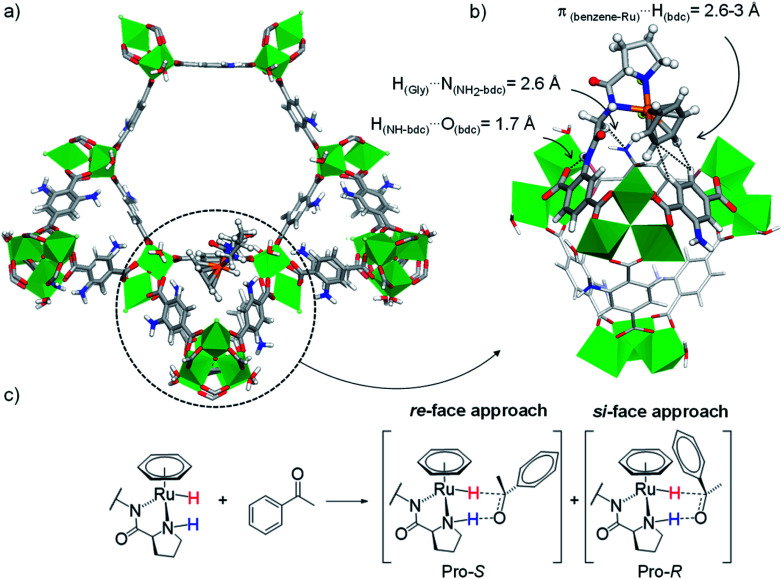
(a) Favoured position of the (benzene)Ru(Gly–Pro) graft in MIL-101-NH_2_ identified from MD/DFT calculations (see ESI, Fig. S2,† model A), viewed along the *c* axis. (b) Detailed re-orientated view showing the (benzene)Ru(Gly–Pro) graft stabilized through H_(Gly)_⋯N_(NH_2_-bdc)_ and π_(benzene-Ru)_⋯H_(bdc)_ interactions with the MOF. (c) ATH reaction of an aromatic ketone with a Noyori-type molecular catalyst: Pro-*S* and Pro-*R* complexes are formed upon the *re*-face and *si*-face approaches of the substrate, respectively. Color code: Al (green), Ru (orange), Cl (pale green), N (blue), O (red), C (grey), H (white).

For these DFT calculations, large finite-size models containing the entire hexagonal window and three ST vicinal to the (benzene)Ru(Gly–Pro) graft were required to mimic the MOF's environment around the catalytic site. Overall, the most favourable model for the (benzene)Ru(Gly–Pro) graft identified in this process (Fig. S2,† model A) exhibits its benzene(Ru) component oriented towards the MOF's dicarboxylate (bdc) linker of a neighboring ST, as illustrated in Fig. 3a and b. The graft is stabilized through π_(benzene-Ru)_⋯H_(bdc)_ interactions with the hydrogens of the MOF's linker and a hydrogen-bond type H_(Gly)_⋯N_(NH_2_-bdc)_ interaction (2.6 Å). MD simulations performed on the periodic counterpart of this model show the persistence of this positioning at room temperature, which was then selected as a starting point for all subsequent computations. To characterize the stereochemistry of the (benzene)Ru(Gly–Pro) catalytic complex, the *R* (rectus)/*S* (sinister) notation was adopted along the N_Pro_–C_Pro_–N_Gly_–Ru sequence of asymmetric atoms noted with asterisks in Fig. 1c. The envisioned ATH reaction of an aromatic ketone to give the corresponding chiral alcohol requires that two hydrogen atoms, *i.e.* the hydridic Ru–H of the metal center and the protic N_Pro_–H of the proline, are in *cis* position to allow their transfer to the C and O atoms, respectively, of the substrate's C=O bond in order to yield the *R*- and/or *S*-alcohols (Fig. 3c and S3†).^37^

This mechanistic requirement implies that only a subset of N_Pro_–C_Pro_–N_Gly_–Ru configurations of the graft may be functional towards the ATH reaction, whereby the two Ru and N_Pro_ atoms should possess identical *R* (or *S*) configurations (*i.e.* either *S*_N(Pro)_, *S*_Ru_ or *R*_N(Pro)_, *R*_Ru_), allowing to reduce the number of the computationally explored stereoisomers from 16 to 8.

Two series of four stereoisomers that are potentially catalytically active for ATH may thus be distinguished that differ by the configuration of their proline, *i.e.* possessing either *S* or *R* configured C_Pro_. The two series correspond to enantiomeric sets, where the four *S*_C(Pro)_- and four *R*_C(Pro)_-containing stereoisomers are mirror images of each other (for instance, *S*_N(Pro)_–*S*_C(Pro)_–*R*_N(Gly)_–*S*_Ru_, and *R*_N(Pro)_–*R*_C(Pro)_–*S*_N(Gly)_–*R*_Ru_). Notably, this analysis allowed for a further reduction of the number of configurations of the graft to be computed. Only the four l-proline containing diastereoisomers are thus discussed below with respect to the ATH reaction in the (benzene)Ru@Al-MIL-101-NH-Gly-(l)Pro material. We carefully checked that the d-proline-containing enantiomers provided rigorously identical computational results to those obtained with their l-proline-containing counterparts, allowing us to reason our findings solely on the subset of l-proline containing models.

The four configurations of the (benzene)Ru(Gly–Pro) graft, *i.e. S*_N(Pro)_–*S*_C(Pro)_–*R*_N(Gly)_–*S*_Ru_, *S*_N(Pro)_–*S*_C(Pro)_–*S*_N(Gly)_–*S*_Ru_, *R*_N(Pro)_–*S*_C(Pro)_–*R*_N(Gly)_–*R*_Ru_ and *R*_N(Pro)_–*S*_C(Pro)_–*S*_N(Gly)_–*R*_Ru_, were computed at the DFT-D3 level using the most favourable anchorage position identified above, noted as variants 1, 2, 3 and 4, respectively (Table 2 and ESI, Section 1.3.1†). The DFT calculations show that the various configurations of the graft establish recurrent stabilizing interactions with the MOF as illustrated in Fig. 3b for 3 (Fig. S4–S6† for 1, 2 and 4, respectively). In all variants, the amide NH_MOF_ – the anchoring point of the graft – features an intramolecular hydrogen bond (1.7–1.8 Å) with the carboxyl oxygen of the linker. This interaction hinders the rotational freedom around the C_linker_–NH_MOF_ bond, thus constraining the orientation of the planar CO_Gly_–NH_MOF_ peptide bond. The rest of the (benzene)Ru(Gly–Pro) graft systematically adopts the typical piano-stool structure of this well-known class of organometallics.^38^ The Ru-metal center is coordinated to the two nitrogen atoms, NH_Gly_ and NH_Pro_, of the –Gly–Pro dipeptide whereby the rotational freedom of the proline around the C_Gly_–C_Pro_ bond is also prohibited. This contrasts with the relative rotational freedom observed in the ruthenium-free MOF–Gly–Pro functionalized framework.^22^ Key short-range interactions with the MOF host stabilize the entire (benzene)Ru(Gly–Pro) graft. Typically, π-type interactions between the benzene ring of the (benzene)Ru moiety and the aromatic hydrogen atoms of the MOF's linker occur in a recurrent fashion in 2, 3 and 4, with π_(benzene-Ru)_⋯H_(bdc)_ distances in the 2.6–3.3 Å range. Additional H_(Gly)_⋯N_NH_2_-bdc_ hydrogen-bond type interactions between the –Gly residue and the amino group of the MOF's linker in the 2.5–2.6 Å range are found to stabilize the (benzene)Ru(Gly–Pro) graft in 1, 3 and 4.

**Table 2:** Energetics of acetophenone in complex with (benzene)Ru@MIL-101-NH-Gly-(l)Pro. Interaction energy differences between the two faces of acetophenone in complex with the catalytic graft, whereby *δ*Δ*E*_inter_(*re*–*si*) is calculated in each variant by exposing acetophenone through its *re*- or its *si*-face to the catalytic site (see ESI for details)

Variant	Configuration^a^	*δ*Δ*E*_inter_(*re*–*si*) (kJ mol^−1^)	Expected enantiomer selectivity
1	*S*–***S***–*R*–*S*	−15.7	*S* > *R*
2	*S*–***S***–*S*–*S*	−3.0	*R*, *S*
3	*R*–***S***–*R*–*R*	−18.1	*S* > *R*
4	*R*–***S***–*S*–*R*	−1.6	*R*, *S*

a The *R* (rectus)/*S* (sinister) notation refers to the configuration adopted along the N_Pro_–C_Pro_–N_Gly_–Ru sequence of asymmetric atoms. The *S* configuration of the asymmetric carbon of the (l)Pro is noted in bold.

### Evaluation of the pro-chiral substrate's approach

As a proof-of-concept, we speculated here that the confinement of the catalytic graft into the MOF has further impact with respect to the ATH reaction and promote the stereospecific recognition of a prochiral substrate through the differentiation of its *re*- and *si*-faces. To test this hypothesis, we performed a detailed computational investigation of host–guest interactions between acetophenone, the prochiral ATH substrate, and the (benzene)Ru@MIL-101-NH-Gly-(l)Pro host.

In ATH reaction, the enantioselectivity of Noyori-type Ru^II^(η^6^-arene) molecular complexes is known to build upon a molecular recognition process whereby differently stabilized transition states (TS) are formed upon exposing the prochiral substrate to the Ru-center through its *si*- or its *re*-face, favouring the formation of the *R* product over *S* due to the formation of stabilizing C–H⋯π interactions in the Pro-*R* complex (Fig. 3c and S3†).^39^ Considering here the very large size of the systems of interest – which precludes calculations of TS – we rather considered the adsorption step of acetophenone in the (benzene)Ru@MIL-101-NH-Gly-Pro host.

**Fig. 4:**
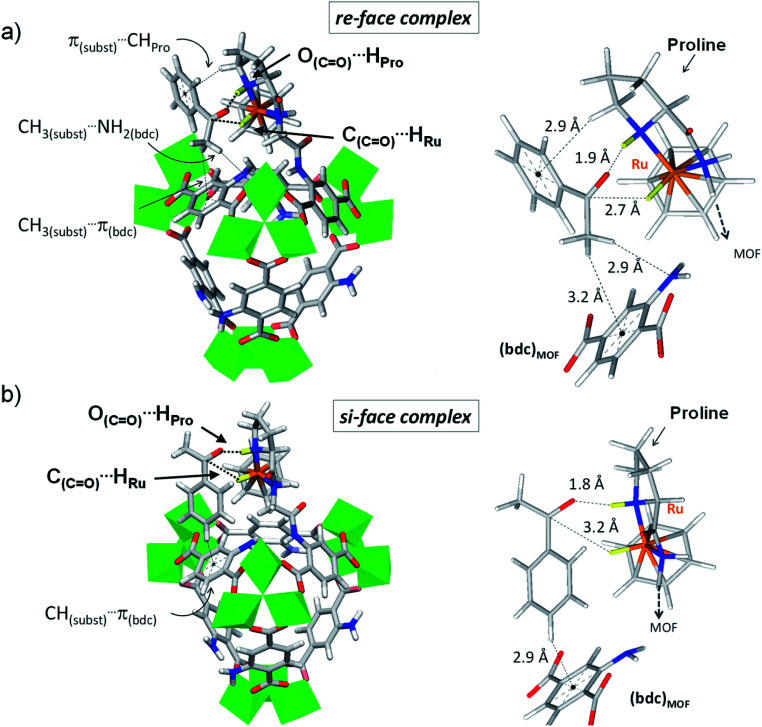
Variant 3 of (benzene)Ru@MIL-101-NH-Gly-(l)Pro in complex with acetophenone computed at the DFT-D3 level. Details of acetophenone exposing its *re*-face (a) and its *si*-face (b) to the catalytic site. Enhanced interactions at the *re*-face with respect to the *si*-face emanate from a shorter C_(C=O)_⋯H_Ru_ distance (2.7 Å *vs.* 3.2 Å) and tighter second-coordination sphere interactions. At the *re*-face, the substrate is also stabilized through π_(substrate)_⋯CH_Pro_ while its methyl group interacts with the MOF linker through CH_3(substrate)_⋯NH_2(bdc)_ and CH_3(substrate)_⋯π _(bdc)_ interactions. Such lateral stabilization cannot occur in the *si*-face complex. Instead, CH_(substrate)_⋯π_(bdc)_ interactions (2.9 Å) with the MOF's linker drift the substrate's C=O bond away from the catalytic Ru center, resulting in a longer C_(C=O)_⋯H_Ru_ distance of 3.2 Å. Color code: Al (green), Ru (orange), Cl (pale green), N (blue), O (red), C (grey), H (white). The H atoms involved in ATH are highlighted in yellow, with the two interactions C_(C=O)_⋯H_Ru_ and O_(C=O)_⋯H_Pro_ required for ATH highlighted with dashed lines.

The potential enantioselectivity of the host for ATH was computationally assessed through a comparison of the affinity of each variant for acetophenone at the Ru catalytic center, as follows. All {(benzene)Ru@MIL-101-NH-Gly-(l)Pro, C_6_H_5_COCH_3_} host–guest complexes were fully optimized at the DFT-D3 level, whereby acetophenone was exposed to the (benzene)Ru(Gly–Pro) catalytic site in each variant 1–4 through its *re*-face and its *si*-face. Host–guest interaction energies, expressed as Δ*E*_inter_(*re*) and Δ*E*_inter_(*si*), were computed through single-point calculations (Δ*E*_inter_(*re*) = *E*_{host,guest-*re*}_ − *E*_{host}_ − *E*_{guest}_). The difference in interaction energy associated to each variant, *δ*Δ*E*_inter_(*re*–*si*) = Δ*E*_inter_(*re*) − Δ*E*_inter_(*si*), provided an estimate of the relative affinity for both faces. Again, all DFT calculations were performed on the large finite-size models (*ca.* 700 atoms) of each variant so that the environment around the catalytic site beyond the second coordination sphere was taken into account. Computed affinity differences are summarized in Table 2. Detailed comparisons between the *re*-face and *si*-face complexes are given in Table S1 and Fig. 4, S7–S9 (see also ESI, Section 1.3.2†).

Interestingly, the DFT calculations show pronounced affinity differences, *δ*Δ*E*_inter_(*re*–*si*), in variants 1 and 3, whereby the *re*-face of acetophenone is favoured over its *si*-face by −15.7 and −18.1 kJ mol^−1^, respectively. The two other variants do not exhibit such marked interaction energy differences, suggesting similar affinities for both faces of the pro-chiral substrate. The favoured interaction of the substrate's *re*-face with respect to its *si*-face in 1 and 3 implies that a molecular recognition of acetophenone may be at play in (benzene)Ru@MIL-101-NH-Gly-(l)Pro. The case of acetophenone in complex with 3 is particularly illustrative (Fig. 4 and Table S1†). The marked preference of 3 for the *re*-face of the substrate originates from the much shorter C_(C=O)_⋯H_Ru_ distance (2.7 Å), hence stronger interactions, than at the *si*-face (3.2 Å). It is overall apparent that the first coordination sphere around the substrate, formed by the protic and hydridic hydrogens of the (benzene)Ru(Gly–Pro) catalytic graft (highlighted in yellow in Fig. 4), allows recognition features of the two faces of acetophenone (see Fig. S7–S9† for 1, 2 and 4 respectively). The pronounced preference for the *re*-face of acetophenone in 1 and 3 prompted us to further analyze host–guest interactions that are more distant to the substrate.

Beyond the first coordination sphere, a series of lateral π-type interactions stabilize acetophenone in all 1–4 variants (Table S1†). They consist in CH/π and NH_2_/π interactions within 2.7–3.3 Å between the substrate's aromatic ring and either the (benzene)Ru moiety or the aromatic linker of the MOF. These interactions occur in a systematic fashion (Table S1†), however with distinctive features between the *si*- and *re*-face complexes. Remarkably, acetophenone in complex in 1 (Fig. S7†) and 3 (Fig. 4) displays a tighter lateral stabilization at the *re*-face than at the *si*-face, in line with the higher affinity differences, *δ*Δ*E*_inter_(*re*–*si*), mentioned above. Both the (benzene)Ru(Gly–Pro) graft and the MOF's linker play a key role, which is particularly illustrative in 3 (Fig. 4). The *re*-face of acetophenone in 3 is stabilized by multiple CH/π interactions with the catalytic graft, *i.e.* π_(substrate)_⋯H_CH(Pro)_ and π_(substrate)_⋯H_(benzene-Ru)_, in addition to interactions with the MOF's linker, *i.e.* CH_3(substrate)_⋯NH_2(bdc)_ and CH_3(substrate)_⋯π_(bdc)_ (Fig. 4a). These features contrast with the fewer ones taking place at the *si*-face (Fig. 4b). Notably, at the *si*-face the substrate is attracted towards the MOF's linker through CH_(substrate)_⋯π_(bdc)_ interactions, which contribute to pull the C=O group away from the catalytic site. This results in a much longer C=O⋯H_Ru_ distance (3.2 Å) than in the *re* complex (2.6 Å), with a potential detrimental impact on the occurrence of ATH reaction at the *si*-face. Conversely, such distinctive features are not observed in 2 or 4, in line with the absence of marked affinity differences, *δ*Δ*E*_inter_(*re*–*si*), as noted above. Accordingly, the analysis of host–guest interactions provides an atomic-scale understanding for the pronounced face differentiation of the prochiral substrate in (benzene)Ru@MIL-101-NH-Gly-(l)Pro catalyst in favour of its *re*-face. They provide a rational basis for the likely favoured formation of *S*-alcohol over that of *R*-alcohol (Table 2).

It is worth highlighting here that the above CH/π lateral host–guest interactions are reminiscent of those reported in Ru^II^(η^6^-arene) Noyori's type molecular catalysts for ATH of α-aryl ketones. Theoretical studies have revealed that the enantioselectivity of such complexes for ATH originates not only from the geometry of the chelate ring formed by the {catalyst, substrate} complex (Fig. S3†) but also from CH/π attractive lateral interactions between CH(η^6^-benzene) and phenyl C(sp^2^) atoms of the aromatic substrate. The latter favours a spatially congested transition state occurring only with one specific face of the prochiral substrate.^39–42^

The stereoselectivity revealed here is specifically dependent of the MIL-101 macroligand's structure supporting the Ru-molecular catalyst, and does not have equivalent in any homogeneous systems reported so far.

When compared to Ru(η^6^-arene) molecular catalysts, the (benzene)Ru@MIL-101-NH-Gly-(l)Pro solid provides peripheral stabilization of the prochiral substrate, whereby both the MOF's linker and the dipeptide graft are involved and allow the differentiation of the two faces of acetophenone.

We thus speculate that the face differentiation of the prochiral ketone in (benzene)Ru@MIL-101-NH-Gly-(l)Pro or in (benzene)Ru@MIL-101-NH-Gly-(d)Pro catalyst for ATH reaction originates not only from the local chirality of the hosted ruthenium catalyst but also from the MOF macroligand itself. We have verified the computational equivalence of the DFT results regarding the (d)Pro-containing host whereby a reversed stereoselectivity is predicted. We anticipate that the face differentiation in (benzene)Ru@MIL-101-NH-Gly-(d)Pro lead to a reversed enantioselectivity in the ATH reaction of acetophenone in favour of the *R*-alcohol product. The correlation between the proline's configuration (with its *R* or *S* carbon) and that of the favoured product (*S* or *R*, respectively) in ATH is indeed established and perfectly in line with the experimental results.

Although calculations omit solvent and solvated ionic species, such as counter anions that are present in the real catalytic medium, our experimental assessments are validating the DFT calculations which predict a reversed enantioselectivity of the molecular catalyst (benzene)Ru(L^2^) when compared to its MOF-supported counterpart (Fig. S25, S26, Table S1 and Section 5.4 in ESI†). Altogether, these results suggest that the inversion of enantioselectivity between the homogeneous and heterogeneous catalysts most likely arises from the steric constraints imposed by the confinement of the chiral catalyst within the MOF's pore. As identified at the DFT level, the latter plays a key role in allowing, amongst others, stereoselective CH/π host–guest interactions with the prochiral acetophenone substrate, in favor of its complexation at the *re*-face in the (l)proline-containing catalyst. Such lateral stabilization cannot occur in the molecular catalyst (benzene)Ru(L^2^) whereby the *si*-face complex and the related *R*-alcohol product are favored.

Computations show that subtle host–guest interactions are at play in the (benzene)Ru@MIL-101-NH-Gly-(l)Pro catalyst whereby the differentiation of the substrate's faces at the adsorption step allows predicting the favored formation of the *R* product, in full agreement with the experimental observations. The very low selectivity of the molecular prolinamide catalyst might arise from the weak constraints of the chiral ligand on the substrate, in which the selectivity relies on the sole CH/π interactions (Noyori-type) between the (arene)ruthenium and the ketone. In contrast, the host–guest interactions imposed by the MOF framework around the catalytic graft – involving its aromatic bdc linker and the proline – induce specific substrate positioning at the origin of the superior enantioselectivity of the MOF-supported (benzene)Ru(Gly–Pro) catalyst.

## Conclusions

In conclusion, the Gly–Pro functionalization of the MIL-101, used as a macroligand to heterogenize a chiral Noyori type Ru-based molecular catalyst, was found to promote enantioselectivity in the asymmetric transfer hydrogenation of acetophenone to phenylethanol. When compared to its homogenous molecular analogue, the chiral ruthenium complex confined within the MOF macroligand allows a threefold enhanced and reversed selectivity in the production of phenylethanol. Furthermore, we successfully addressed the challenge of computationally generating a series of large hybrid MOF structures embedding flexible chiral peptide graft as ligand for organometallics. The systematic computational evaluation unveiled how host–guest interactions within the MOF and beyond the first coordination sphere of the ruthenium are at the origin of the face-differentiation of acetophenone favouring its *re*-face approach over its *si*-face, when using (l)-proline, successfully modelling an excess of the *S*-alcohol over Ru-catalyzed ATH reaction.

The excellent match between the predicted outcomes and the experimentally obtained enantiomeric excess provides a robust atomic-level rationale for the observed products selectivity. The combined computational and experimental findings highlight the crucial role of the MOF as both a macroligand and a supramolecular scaffold to promote the enantioselectivity of the ruthenium chiral catalyst. More generally, the ability to provide molecular-level rationale of structure–reactivity relationship in heterogeneous asymmetric transformation at the solid's interface within multifunctional porous hybrids opens new routes to develop predictive frameworks in the design of heterogeneous catalysts.

## Conflicts of interest

There are no conflicts to declare.

## Supplementary Material

SC-011-D0SC03364B-s001

SC-011-D0SC03364B-s002

SC-011-D0SC03364B-s003

SC-011-D0SC03364B-s004

SC-011-D0SC03364B-s005

SC-011-D0SC03364B-s006

SC-011-D0SC03364B-s007

SC-011-D0SC03364B-s008

SC-011-D0SC03364B-s009

SC-011-D0SC03364B-s010

SC-011-D0SC03364B-s011

SC-011-D0SC03364B-s012

SC-011-D0SC03364B-s013

SC-011-D0SC03364B-s014

SC-011-D0SC03364B-s015

SC-011-D0SC03364B-s016

SC-011-D0SC03364B-s017
