# Supplementary material for: Synthetic and computational assessment of a chiral metal–organic framework catalyst for predictive asymmetric transformation
Source: Chem Sci. 2020 Aug 6;11(33):8800–8. doi: 10.1039/d0sc03364b (PMC8163446; doi:10.1039/d0sc03364b)
Supplement: SC-011-D0SC03364B-s017 [file SC-011-D0SC03364B-s017.pdf]

Electronic Supplementary Material (ESI) for Chemical Science.
This journal is © The Royal Society of Chemistry 2020

Electronic Supplementary Information

Synthetic and computational assessment of a chiral metal-organic framework catalyst for predictive asymmetric transformation

Jerome Canivet,*[,1] Elise Bernoud,[1] Jonathan Bonnefoy,[1] Alexandre Legrand,[1] Tanya K. Todorova,[2] Elsje Alessandra Quadrelli,[3] and Caroline Mellot-Draznieks,*[,2]

[1] Univ. Lyon, Université Claude Bernard Lyon 1, CNRS, IRCELYON UMR 5256, 2 Av. Albert Einstein, 69626 Villeurbanne, France; [2] Laboratoire de Chimie des Processus Biologiques, Collège de France, Sorbonne Université, CNRS UMR 8229, 11 Place Marcelin Berthelot, Paris 75231 Cedex 05, France;[3] Univ. Lyon, Université Claude Bernard Lyon 1, CNRS, C2P2 UMR 5265, 43 Bvd du 11 Novembre 1918, 69616 Villeurbanne, France.

Corresponding authors:

jerome.canivet@ircelyon.univ-lyon1.fr

caroline.mellot-draznieks@college-de-france.fr

1. Computational Details……… 5
1.1. Simulation of (benzene)Ru@MIL-101-NH-Gly-Pro periodic models……… 5
1.1.1. Preparation of the aminated MIL-101-NH_2 crystal structure……… 5
1.1.2. Preparation of the (benzene)Ru(Gly-(*L*)Pro) complex……… 6
1.1.3. Construction of (benzene)Ru@MIL-101-NH-Gly-(*L*)Pro periodic models and Molecular Dynamics……… 6
1.1.4. DFT calculations of (benzene)Ru@MIL-101-NH-Gly-(*L*)Pro finite-size models……… 7
1.2. Acetone in Noyori-type complexes……… 9
1.3. Stereoisomers of (benzene)Ru@MIL-101-NH-Gly-(*L*)Pro and their complex of with acetophenone……… 9
1.3.1. DFT calculations of (benzene)Ru@MIL-101-NH-Gly-(*L*)Pro stereoisomers……… 10
1.3.2. Acetophenone in complex with (benzene)Ru@MIL-101-NH-Gly-(*L*)Pro stereoisomers……… 13
2. Materials synthesis……… 16
2.1 General remarks……… 16
2.2 Peptide coupling……… 16
2.3 Ruthenium coordination……… 17
3. Materials characterizations……… 18
3.1 Powder X-ray diffraction……… 18
3.2 Nitrogen physisorption……… 19
3.3 Electron microscopy……… 21
4. **Synthesis of L^1 and L^2 molecular ligands** ……… 22
5. **Catalytic ketone transfer hydrogenation**……… 25
5.1 Heterogeneous catalysis conditions……… 25
5.2 Homogeneous catalysis conditions……… 25
5.3 HPLC analysis……… 26
5.4 DFT computations with the {(benzene)Ru(L^2), acetophenone} molecular comple……… 29
6. **References**……… 31

Figures & Tables

Figure S1. MIL-101-NH_2 crystal structure……………………………………………………5

Figure S2. Exploring the positioning of the (benzene)Ru(Gly-Pro) complex grafted in the MIL-101 solid. …..……………………………………………………………………………...…… 7

Figure S3. Interactions at play in Noyori-type molecular complexes upon ATH reaction.…… 9

Figure S4. View along the *c* axis of the (benzene)Ru@MIL-101-NH-Gly-(*L*)Pro variant 1...10

Figure S5. View along the *c* axis of the (benzene)Ru@MIL-101-NH-Gly-(*L*)Pro variant 2...11

Figure S6. View along the *c* axis of the (benzene)Ru@MIL-101-NH-Gly-(*L*)Pro variant 4...12

Figure S7. Variant 1 *(S-S-R-S)* in complex with acetophenone computed at the DFT-D3 level………………………………………………………………………………………......13

Figure S8. Variant 2 (*S-S-S-S*) in complex with acetophenone computed at the DFT-D3 level……………………………………………………………………………………...……14

Figure S9. Variant 4 (*R-S-S-R*) in complex with acetophenone computed at the DFT-D3 level……..…15

Figure S10. ^{1}H NMR spectrum of MIL-101-NH-Gly-Pro dissolved in HF-H_2O/dmso d[6] solution……………………………..…………………………………..……………….…..……17

Figure S11. Power X-ray diffraction patterns of functionalized MIL-NH_2……………..…….. 18

Figure S12. Nitrogen physisorption isotherms of the (benzene)Ru@MIL-101-NH-Gly-Pro solids …………...………..........................…………………………………………....……………..……19

Figure S13. BET plot calculated from the N_2 physisorption isotherms of the (benzene)Ru@MIL-101-NH-Gly-Pro solids…...20

Figure S14. ^{1}H NMR spectrum of (benzene)Ru@MIL-101-NH-Gly-(*L*)Pro dissolved in HF-H_2O/dmso d[6] solution………………………………………………………..………………….21

Figure S15. TEM micrographs of (benzene)Ru@MIL-101-NH-Gly-(*L*)Pro ………...…...…….21

Figure S16. ^{1}H NMR spectrum of the molecular ligand Boc-L^2……………………......……… 23

Figure S17. ^{1}H NMR spectrum of the molecular ligand L^2……..…………………...…………..…24

Figure S18. ^{13}C NMR spectrum of the molecular ligand L^2 ……...……….…..….….…………….. 24

Figure S19. Typical chromatogram and retention times obtained for the acetophenone ATH reaction catalyzed by $(C_6H_6)Ru(L^1)Cl_2$…………………………………………..………………… 26

Figure S20. Typical chromatogram and retention times obtained for the acetophenone ATH reaction catalyzed by $(C_6H_6)Ru(L^2)Cl_2$……………………………………..……………….…….26

Figure S21. Typical chromatogram and retention times obtained for the acetophenone ATH reaction catalyzed by (benzene)Ru@MIL-101-NH-Gly-(*L*)Pro.. 27

Figure S22. Typical chromatogram and retention times obtained for acetophenone ATH reaction catalyzed by (benzene)Ru@MIL-101-NH-Gly-(*D*)Pro...27

Figure S23. Acetophenone ATH reaction catalyzed by (benzene)Ru@MIL-101-NH-Gly-(*L*)Pro…...……………………………………….……………..…28

Figure S24. Recycling experiments for acetophenone ATH reaction catalyzed by (benzene)Ru@MIL-101-NH-Gly-(*L*)Pro ……………………………………..……………….....… 28

Figure S25. *Re*-face approach towards $(C_6H_6)Ru(L^2)Cl_2$ molecular catalyst……...……………..29

Figure S26. *Si*-face approach towards $(C_6H_6)Ru(L^2)Cl_2$ molecular catalyst……………………29

Table S1. Structural and energetic features of acetophenone in complex with (benzene)Ru@MIL-101-NH-Gly-(*L*)Pro.. 30

1. Computational details

1.1. Simulations of (benzene)Ru@MIL-101-NH-Gly-Pro periodic models

The crystal structure of Al-MIL-101, namely $Al_3Cl(H_2O)_2O[(O_2C)\text{-}C_6H_4\text{-}(CO_2)]_3$, was derived from the atomic coordinates previously reported in our X-ray diffraction and computational work, by replacing Cr with Al atoms.[1] The MIL-101 crystal structure is composed of Al_3O trimers, which are linked by 1,4-benzenedicarboxylic (bdc) acids generating supertetrahedra (ST) as the smallest cages (Figure S1, left). As such, MIL-101 adopts the augmented Mobil Thirty-Nine (MTN) zeotype 1. Each ST can be formally described by Al_3O trimers that occupy each of the four corners, while the 1,4-bdc organic linkers occupy the six edges. Figure S1 represents the various building components of MIL-101's crystal structure. The corner-sharing of the ST results in two types of mesoporous cages connected through 5-membered rings. The middle-size cages emanate from the corner-sharing of 20 ST forming a dodecahedron possessing exclusively 5-membered rings (highlighted in yellow in **Figure S1**). The largest cages are composed of 28 ST and exhibit both 5-membered and 6-membered rings (highlighted in blue in Figure S1). Overall this MOF exhibits a complex crystal structure with more than 14 000 atoms per unit cell and an extremely large cell volume (a=b=c=88.7 Å, V ~700 000 Å^3), making DFT calculations particularly challenging and prohibiting their use in a routine manner.

1.1.1. Preparation of the aminated MIL-101-NH_2 crystal structure. In a preliminary step, the periodic crystal structure of MIL-101 was recast to its primitive cell, thereby reducing the cell volume and the number of atoms (V ~170 000 Å^3, ~ 3 700 atoms) and facilitating the subsequent grafting of the catalytic ruthenium-based complex. Each bdc linker was functionalized with an –NH_2 group in a random fashion, *i.e.* by replacing one single H atom of each phenyl ring with one –NH_2 group. We have shown in a previous study on MIL-68 that such functionalization of bdc with amino groups does not follow any specific pattern, suggesting that all four hydrogen atoms of the bdc's aromatic ring can be considered as equally possible for –NH_2 functionalization.[2] In addition, the coordination of Al centers in each Al_3O trimer was completed so as to match the reported experimental chemical composition,[3] *i.e.* one Cl atom was added to one Al ion of each trimer, while a water molecule was added on the other two Al ions. The geometry of this Al-MIL-101-NH_2 structure was finally optimized at the DFT-D3 level using VASP[4] (see section 1.1.4).

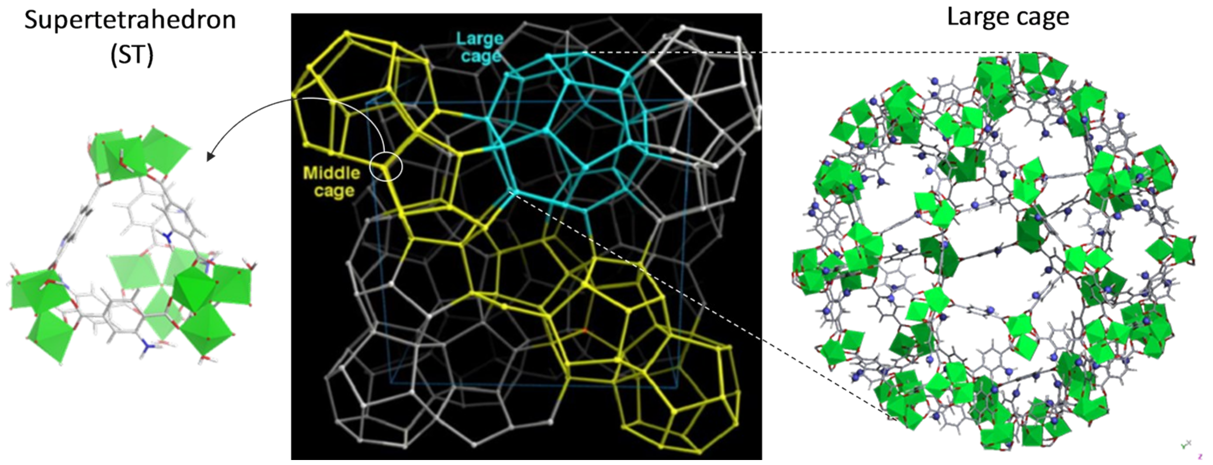

Figure S1. **MIL-101-NH$_2$ crystal structure.** Left: Polyhedral representation of a hybrid ST constitutive of MIL-101-NH$_2$ crystal structure; each ST combines bdc-NH$_2$ linkers on edges positions and inorganic trimers made of Al-octahedra at each of the four corners. Color code: Al (green), O (red), C (grey), N(blue), and H (white). Middle: Schematic representation of Al-MIL-101-NH$_2$ crystal structure, where ST are represented as corner-sharing nodes (linear stick), allowing recognizing the MTN zeotype structure. Cages composed exclusively of 5-membered rings are highlighted in yellow, while a large cage exhibiting both 5- and 6-membered rings is shown in blue. Right: polyhedral and stick representation of a large cage of MIL-101-NH$_2$.

1.1.2. Preparation of the (benzene)Ru(Gly-Pro) complex. The initial conformation of the Gly-Pro dipeptide was taken from our previous study of the peptide-functionalized MIL-68-Gly-Pro, using the (*L*)proline.[2] The (benzene)Ru(Gly-Pro) complex was further constructed by coordinating the [(benzene)RuH] component to the two nitrogen atoms of the Gly-Pro dipeptide. The acetonitrile molecule was replaced by a hydride in order to mimic the active species at play during the catalytic event. The geometry was further optimized using the *uff* forcefield.[5] The (benzene)Ru complex adopts the typical piano-stool structure of this well-known class of organometallics and was further grafted in MIL-101-NH$_2$ periodic model constructed above.

1.1.3. Construction of (benzene)Ru@MIL-101-NH-Gly-(*L*)Pro periodic models and Molecular Dynamics. This step consisted in building various initial periodic constructs of the **(benzene)Ru@MIL-101-NH-Gly-(*L*)Pro** solid in order to explore the positioning of the (benzene)Ru(Gly-Pro) graft and identify the most likely one within the MIL-101 host. Having in mind the complexity of MIL-101's crystal structure, the functionalization was restricted to a single (benzene)Ru(Gly-Pro) catalytic complex per unit cell within a 6-membered ring of a large cage. We hypothesized here that the grafting of the Gly-Pro dipeptide may occur experimentally more easily in a large cage than in a middle-size cage, and more easily in a 6-membered ring rather than in a 5-membered ring as a result of reduced steric hindrance. This is also relevant for assuring accessibility of the substrate to the catalytic graft. Four initial periodic models (model-1,-2,-3,-4) of **(benzene)Ru@MIL-101-NH-Gly-(*L*)Pro** were thus constructed exploring various initial grafting positions of the (benzene)Ru(Gly-Pro) complex. For simplicity, we used in this step the R_{NPro}-S_{CPro}-R_{NGly}-R_{Ru} configuration for the (benzene)Ru(Gly-Pro) complex. The four initial periodic constructs were subjected to geometry optimizations

followed by molecular dynamics (MD) simulations allowing an extensive sampling of the conformational space. Constrained by the very large number of atoms, this step utilized the so-called universal force field (*uff*)[5] which provided suitable parameters for all atoms, in particular Ru, Al and Cl. The MD runs were performed under the constraints of fixed cell parameters while allowing all atoms to relax. The non-bonded interactions between pairs of atoms were represented by Lennard-Jones potentials, calculated in real space within relevant cutoffs, while the Coulombic term was evaluated by the Ewald summation, which describes the electrostatics. The atomic charges for each model were calculated by the charge-equilibrium method. 50 ns NVT MD simulations (constant number of particles, volume, and temperature) were carried out on each periodic model at 298 K, with an integration time step set to 0.1 fs. A subset of periodic models including equilibrium positions and less stable ones were subsequently extracted from the MD simulations and optimized at the density functional theory (DFT) level, as detailed below.

1.1.4. DFT calculations on (benzene)Ru@MIL-101-NH-Gly-(*L*)Pro finite-size models. Considering the extremely large size of the periodic systems generated above, DFT calculations were performed on finite size models (<700 atoms) cleaved from the periodic models generated over the MD simulations. The finite size models were designed to maintain an overall neutral charge and to preserve the host-guest interactions between the catalytic graft and the MOF occurring in the periodic solid. For this purpose, the finite size models were constructed so that the functionalized catalytic linker is embedded in its 6-membered ring window and surrounded with three neighboring STs, as illustrated in Figure S2. In order to ensure electroneutrality, the models were terminated with hydrogen atoms in place of the pendant linkers. All finite-size models considered here possess the same chemical formula, namely $C_{211}H_{129}N_{23}O_{182}Al_{36}Cl_{12}Ru$ and were computed in a box with the 40 Å × 40 Å × 25 Å dimensions. The calculations were performed with the Vienna Ab-initio Simulation Package VASP[5] and allowed to determine the precise geometries and relative energies. The four most stable positions of the catalytic graft identified in this process within the **(benzene)Ru@MIL-101-NH-Gly-(*L*)Pro** material (referred to as A, B, C, D) are represented in **Figure S2**.

The Perdew-Burke-Ernzerhof (PBE) exchange-correlation functional was used[6] along with the semi-empirical vdW method of Grimme DFT-D3.[7] A plane-wave basis set with an energy cutoff of 400 eV was employed for the geometry optimizations utilizing PAW pseudopotentials on all atoms to describe the electron-ion interactions.[8,9] For Ru, the 4p semi-core states were treated as valence (Ru_pv). Atomic positions were optimized until the forces on all atoms were smaller than 0.02 eV $Å^{-1}$. The Brillouin zone was sampled only at the Γ-point.

Figure S2 compares the various orientations of the catalytic graft and their relative energies taking the most stable model A given as a reference. Model A exhibits the benzene(Ru) component of the (benzene)Ru(Gly-Pro) graft oriented towards the MOF's dicarboxylate (bdc) linker of a neighboring ST. This orientation is notably stabilized through π–type interactions between the Ru-benzene ring and hydrogen atoms of the bdc linker ($\pi_{(benzene)}\cdots H_{(bdc)}$ = 2.6 to 3 Å) and short $H_{Gly}\cdots NH_2$ interactions (2.6 Å). MD calculations performed on the parent periodic structure of model A show the persistence of these interactions with the MOF at room temperature. This model A was thus selected as a starting structure for all subsequent

computations and to further explore various possible configurations of the (benzene)Ru(Gly-Pro) graft (*vide infra*).

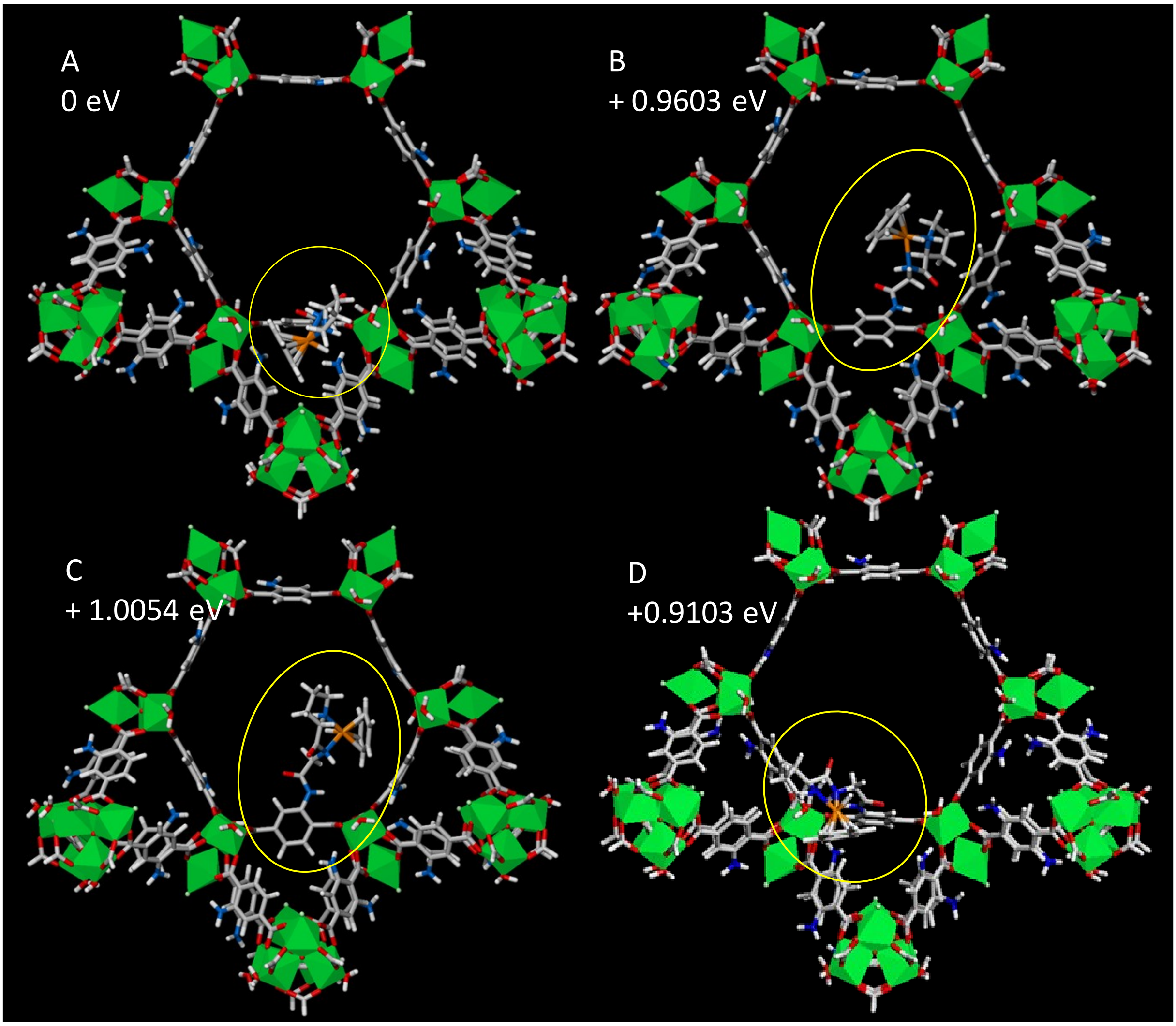

Figure S2. Exploring the positioning of the (benzene)Ru(Gly-Pro) complex grafted in the MIL-101 solid. Various periodic **(benzene)Ru@MIL-101-NH-Gly-(*L*)Pro** constructs were submitted to MD and finite-size models extracted for further DFT-level calculations. The relative energies of the four finite-size models, A, B, C and D, are compared here at the DFT-D3 level, given in eV relative to the most stable model A. All **(benzene)Ru(Gly-Pro)-**containing models computed at this stage possess the same configuration (R_{NPro}-S_{CPro}-R_{NGly}-R_{Ru}) of the graft.

1.2. Acetophenone in Noyori-type complexes

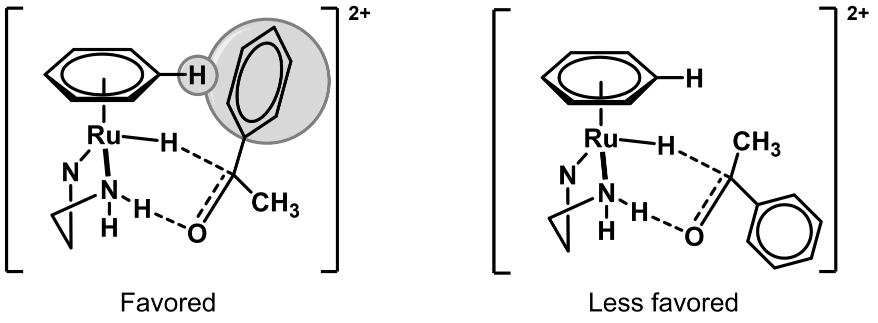

Figure S3. Interactions at play in Noyori-type molecular complexes upon ATH reaction. Pro-S (right) and Pro-R (left) complexes are formed via the *re*-face and *si*-face approaches of the substrate, respectively**.** Stabilizing role of CH-π interactions in the substrate orientation in the Pro-*R* complex (left) as compared to the Pro-*S* complex (right) during asymmetric hydrogen transfer via a metal-ligand mechanism as proposed by Noyori *et al.* [10]

1.3. Stereoisomers of (benzene)Ru@MIL-101-NH-Gly-(*L*)Pro and their interactions with acetophenone

First, we explored the four catalytically active configurations of the *L*-proline containing graft, *i.e.* S_{NPro}-S_{CPro}-R_{NGly}-$S_{\mathbf{Ru}}$ (variant **1**), S_{NPro}-S_{CPro}-S_{NGly}-$S_{\mathbf{Ru}}$ (variant **2**), R_{NPro}-S_{CPro}-R_{NGly}-$R_{\mathbf{Ru}}$ (variant **3**) and R_{NPro}-S_{CPro}-S_{NGly}-$R_{\mathbf{Ru}}$ (variant **4**). These variants all contain an *L*-proline, *i.e.* an *S*-configured C_{Pro}, and were first geometry optimized at the DFT-D3 level using the favorable orientation identified above in model A as a starting positioning. Second, for each of the four configurations of the graft, we performed series of D3-DFT calculations on the entire {**(benzene)Ru@MIL-101-NH-Gly-(*L*)Pro**, aromatic ketone} complex to investigate the affinity of the catalytic graft for the ketone substrate. For each configuration, we considered the two cases whereby the acetophenone substrate is exposed through either its *re*- or its *si*-face to the catalytic graft. In all cases, the {**(benzene)Ru@MIL-101-NH-Gly-(*L*)Pro**, aromatic ketone} complexes were fully optimized at the DFT-D3 level. The interaction energy between the substrate and the MOF macroligand was calculated from the optimized {**(benzene)Ru@MIL-101-NH-Gly-(*L*)Pro**, substrate} complex through three single point calculations as follows:

$$\Delta E_{\text{interaction energy}} = E\{\text{(benzene)Ru@MIL-101-NH-Gly-(}L\text{)Pro, aromatic ketone}\} - E\{\text{(benzene)Ru@MIL-101-NH-Gly-(}L\text{)Pro}\} - E\{\text{aromatic ketone}\}$$

where E{(benzene)Ru@MIL-101-NH-Gly-(*L*)Pro} and E{aromatic ketone} are single point energies of the macroligand and of the substrate, respectively, extracted from the converged {(benzene)Ru@MIL-101-NH-Gly-(*L*)Pro, substrate} finite-size model. The difference in interaction energies between both faces, $\delta\Delta E_{\text{inter}}(re\text{-}si) = \Delta E_{\text{inter}}(re)\text{-}\Delta E_{\text{inter}}(si)$, provided an estimate of the relative affinity of the catalyst for the substrate's *re* face with respect to its *si* face.

1.3.1. DFT calculations of (benzene)Ru@MIL-101-NH-Gly-(*L*)Pro stereoisomers.

- Variant **1** (the configuration of asymmetric atoms is $S_{N(Pro)}$-$S_{C(Pro)}$-$R_{N(Gly)}$-S_{Ru} or *S*-*S*-*R*-*S*):

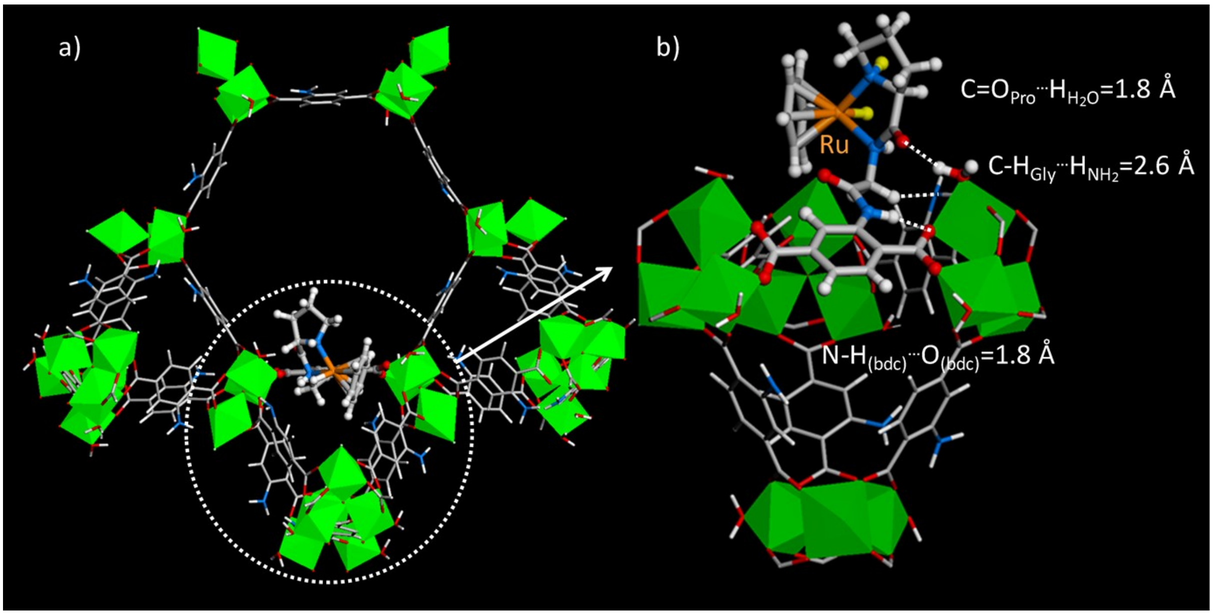

Figure S4. View along the *c* axis of (benzene)Ru@MIL-101-NH-Gly-(*L*)Pro variant 1. Left) View along the *c* axis of **1** as obtained from DFT-D3 calculations. Right) Detailed view of the **(benzene)Ru(Gly-Pro)** catalytic graft and its interactions with the hybrid host framework. Color code: Al (green), Ru (orange), N (blue), O (red), C (grey), H (clear grey). The two hydrogen atoms of the complex involved in the ATH reaction are indicated in yellow.

- Variant **2** (the configuration of asymmetric atoms is $S_{N(Pro)}$-$S_{C(Pro)}$-$S_{N(Gly)}$-S_{Ru} or *S*-*S*-*S*-*S*):

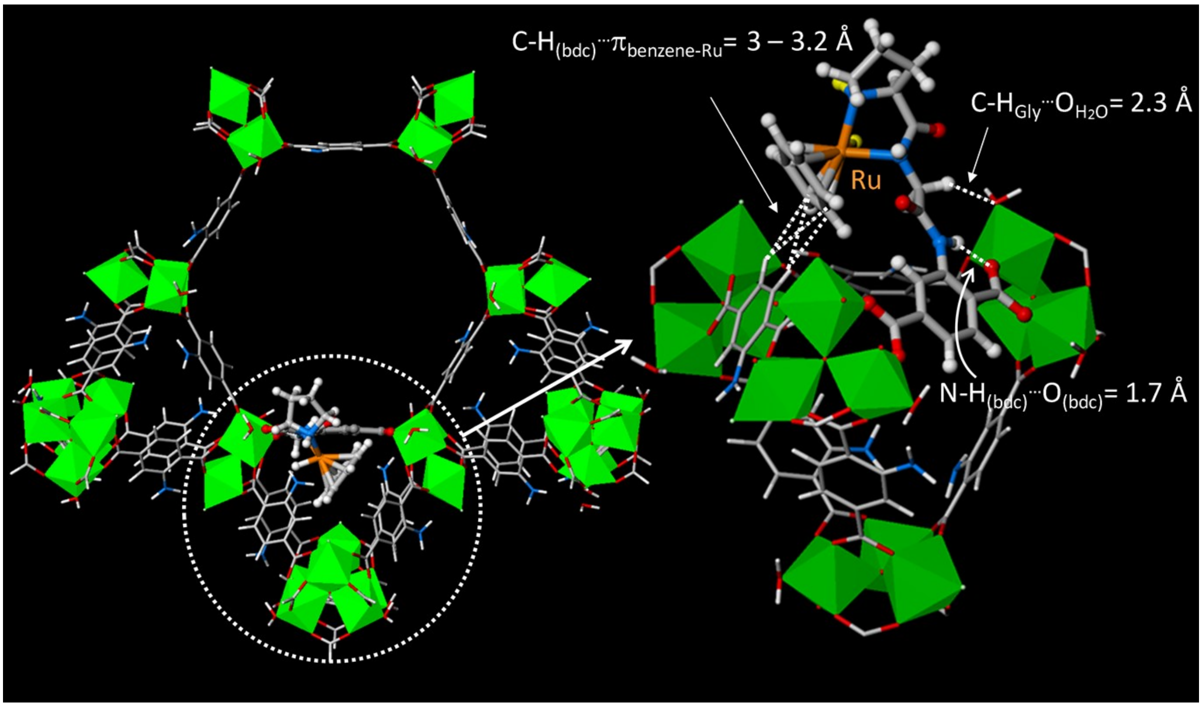

Figure S5. View along the *c* axis of (benzene)Ru@MIL-101-NH-Gly-(*L*)Pro variant 2. Left) View along the *c* axis of **2** as obtained from DFT-D3 calculations. Right) Detailed view the **(benzene)Ru(Gly-Pro)** graft and key interactions with the hybrid framework. See Figure S4 for color coding. The two hydrogen atoms of the complex involved in the ATH reaction are indicated in yellow.

-Variant **3** with the configuration $R_{N(Pro)}$-$S_{C(Pro)}$-$R_{N(Gly)}$-R_{Ru} of asymmetric atoms is shown in Fig. 2 in the main text.

- Variant **4** (the configuration of asymmetric atoms is $R_{N(Pro)}$-$S_{C(Pro)}$-$S_{N(Gly)}$-R_{Ru} or *R*-*S*-*S*-*R*):

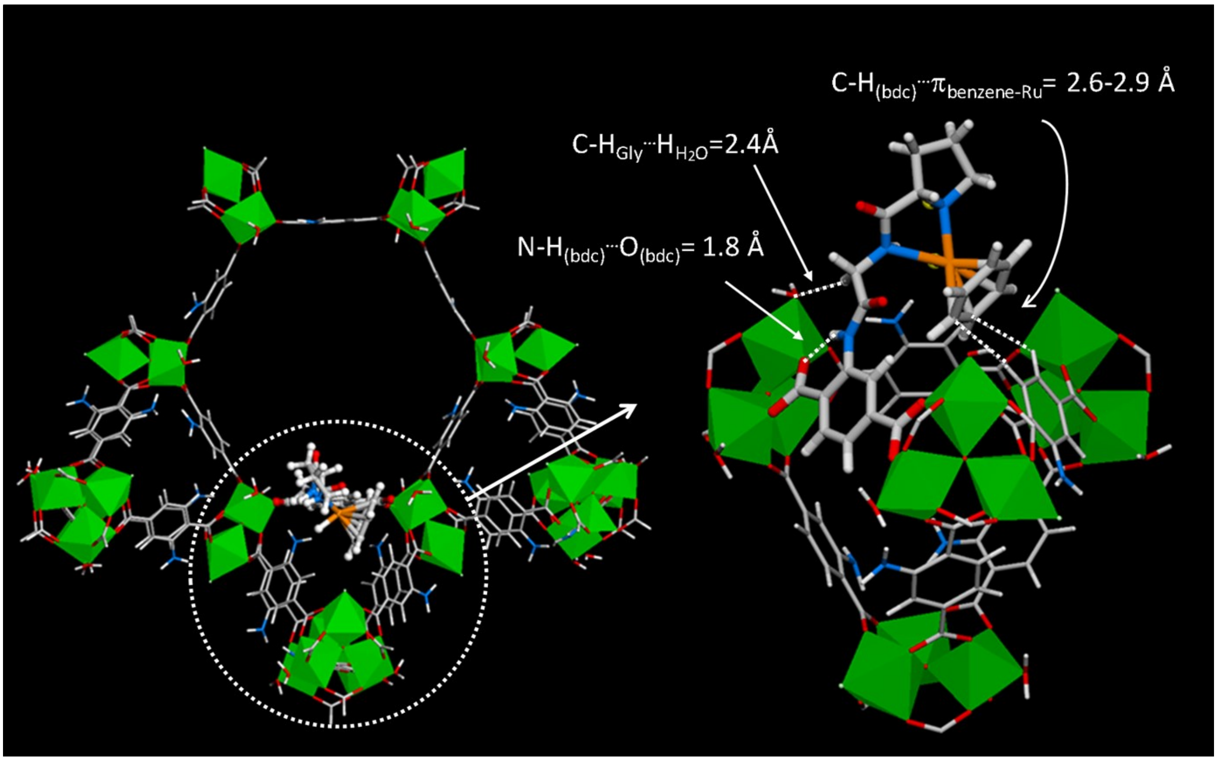

Figure S6. View along the *c* axis of (benzene)Ru@MIL-101-NH-Gly-(*L*)Pro variant 4. Left) View along the *c* axis of **4** as obtained from DFT-D3 calculations. Right) Detailed view the (benzene)Ru(Gly-Pro) graft and key interactions with the hybrid framework. See Figure S4 for color coding. The two hydrogen atoms of the complex involved in the ATH reaction are indicated in yellow.

1.3.2. Acetophenone in complex with (benzene)Ru@MIL-101-NH-Gly-(*L*)Pro stereoisomers

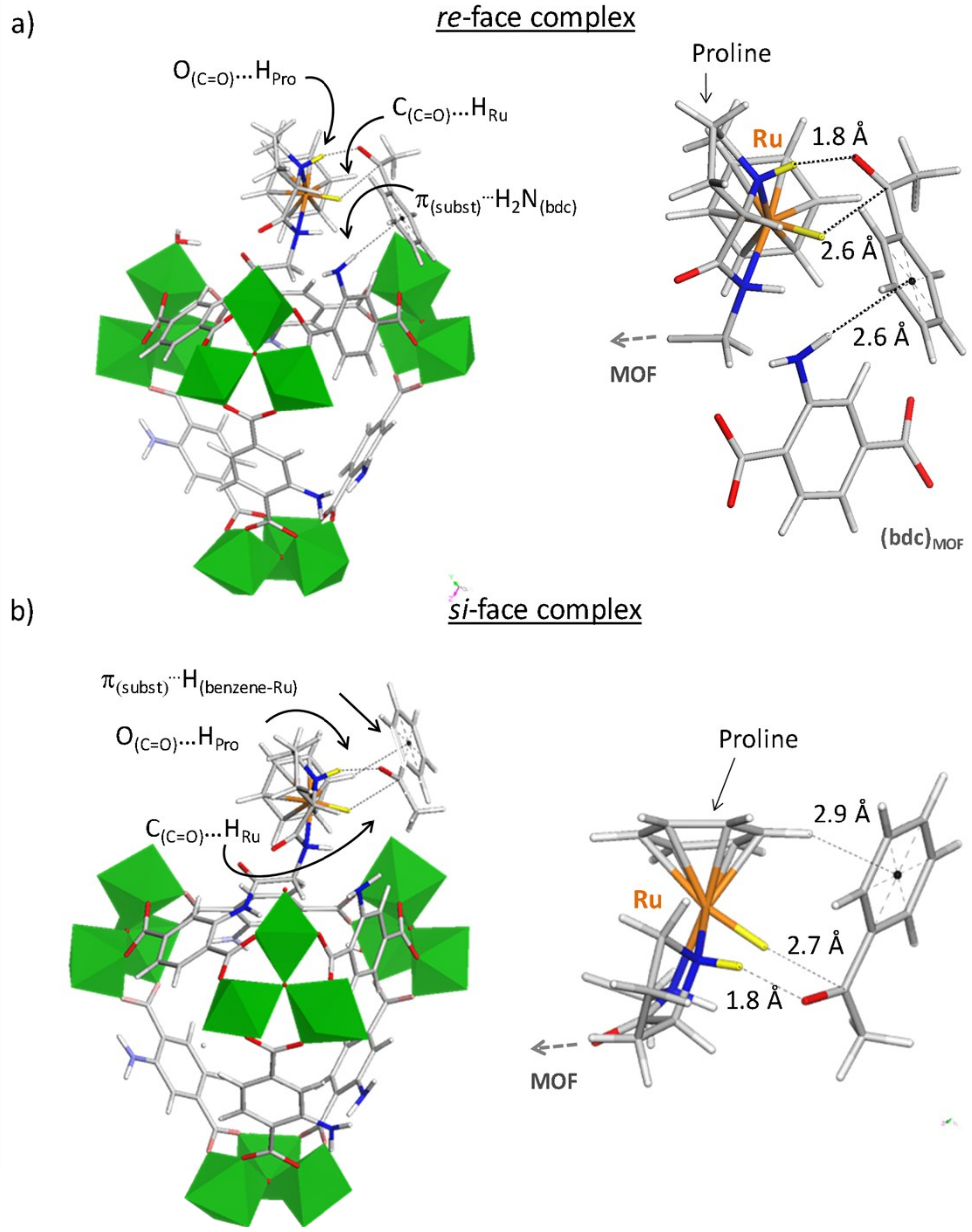

Figure S7. Variant 1 (*S-S-R-S*) in complex with acetophenone computed at the DFT-D3 level. The hydridic H_{Ru} and protic H_{Pro} hydrogen atoms involved in the ATH reaction are highlighted in yellow. Al atoms are depicted as green polyhedra, N is blue, O is red, C is grey, and H is white. The {**(benzene)Ru@MIL-101-NH-Gly-(*L*)Pro**, acetophenone} complexes are compared upon exposing the substrate to the catalytic site through its *re* face (a) and its *si* face (b). The *re*-face is favored with respect to the *si*-face, with a computed interaction energy difference, $\delta\Delta E_{inter}$(*re-si*), of -15.7 kJ.mol^{-1}. **a)** At the *re*-face, the positioning of the substrate's C=O group with respect to the hydridic H_{Ru} allows a slightly shorter C(C=O)…H_{Ru} distance of 2.6 Å than in the *si*-face complex (2.7 Å). Importantly, the amino group of a MOF's linker favors the positioning of the *re*-face of the substrate through a $\pi_{(substrate)}\cdots H_2N_{(bdc)}$ interaction (2.6 Å) with the phenyl C(sp^2) atoms of acetophenone. **b)** The latter lateral stabilization from the MOF cannot occur at the *si*-face of the substrate, whereby no specific role of the MOF's linker is observed. Instead, the *si*-face complex exhibits weaker $\pi_{(substrate)}\cdots H_{(benzene-Ru)}$ interactions between the substrate's aromatic ring and an H atom of the benzene ring (2.9 Å).

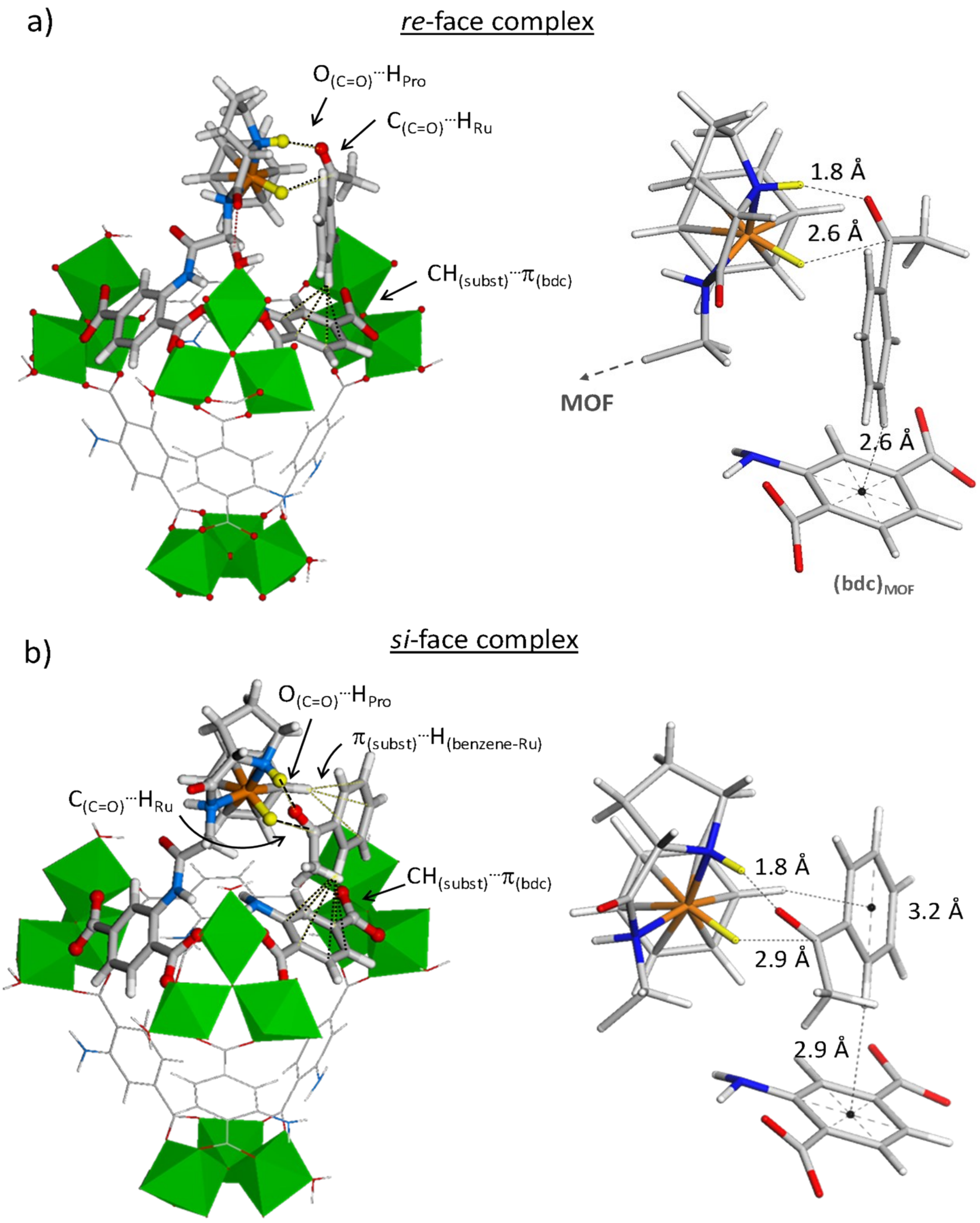

Figure S8. Variant 2 (*S*-*S*-*S*-*S*) in complex with acetophenone computed at the DFT-D3 level. The hydridic H_{Ru} and protic H_{Pro} hydrogen atoms involved in the ATH reaction are highlighted in yellow. The {**(benzene)Ru@MIL-101-NH-Gly-(*L*)Pro**, acetophenone} complexes are compared when acetophenone is exposed to the catalytic site through its *re*-face (a) and its *si*-face (b). The computed interaction energy difference, $\delta\Delta E_{inter}$(*re*-*si*) is estimated ~-3 kJ.mol^{-1}, suggesting a similar stabilization of both faces of the substrate. **a)** At the *re*-face, the positioning of the substrate's C=O group with respect to the hydridic H_{Ru} allows a shorter distance $C_{(C=O)}\cdots H_{Ru}$ (2.6 Å) than at the *si*-face (2.9 Å); the lateral stabilization of the substrate occurs through $CH_{(substrate)}\cdots\pi_{(bdc)}$ interactions (2.6 Å to the bdc's centroid). **b)** Although the *si*-face complex exhibits a longer $C_{(C=O)}\cdots H_{Ru}$ distance than at the *re*-face, it is stabilized by two types of lateral interactions, *i.e.* $CH_{(substrate)}\cdots\pi_{(bdc)}$ and $\pi_{(substrate)}\cdots H_{(benzene\text{-}Ru)}$ interactions. Al atoms are depicted as green polyhedra, N is blue, O is red, C is grey, and H is white.

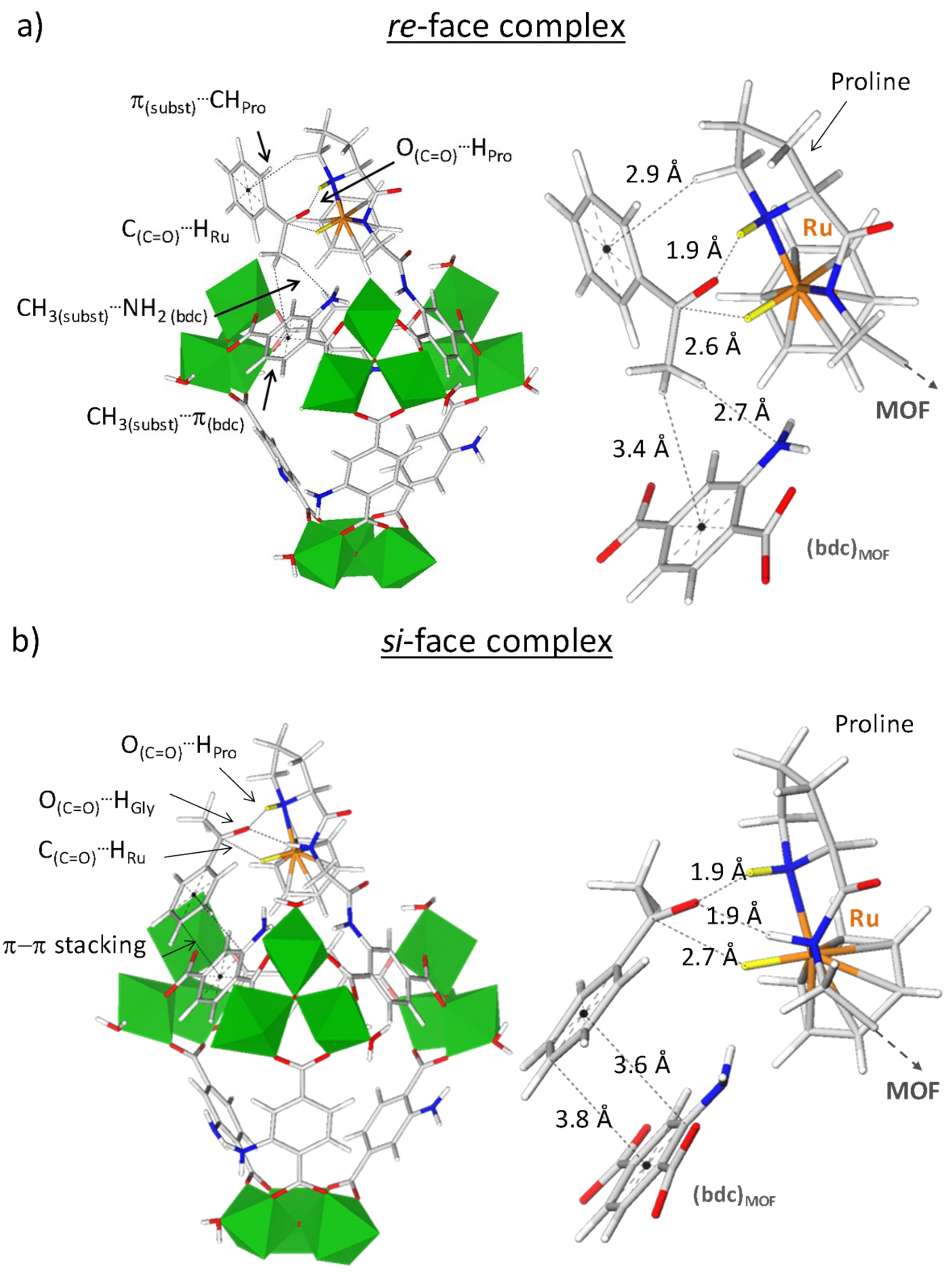

Figure S9. Variant 4 (*R*-*S*-*S*-*R*) in complex with acetophenone computed at the DFT-D3 level. The hydridic H_{Ru} and protic H_{Pro} hydrogen atoms involved in the ATH reaction are highlighted in yellow. Al atoms are depicted as green polyhedra, N is blue, O is red, C is grey, and H is white. The {**(benzene)Ru@MIL-101-NH-Gly-(*L*)Pro**, acetophenone} complexes are compared when acetophenone is exposed to the catalytic center through its *re*-face (a) and its *si*-face (b). The computed interaction energy difference, $\delta\Delta E_{inter}$(*re-si*) is of -1.6 kJ.mol^{-1}, suggesting a similar stabilization of both faces of the substrate. The positioning of the substrate's C=O group with respect to the hydridic H_{Ru} and vicinal protic H_{Pro} hydrogen atom of the proline allows a $C_{(C=O)}\cdots H_{Ru}$ distance of 2.6 Å *vs* 2.7 Å in the *si*-face complex, and an $O_{(C=O)}\cdots H_{Pro}$ distance of 1.9 Å in both cases. The lateral stabilization of the substrate by the MOF is ensured at both faces, by $CH_3\cdots\pi_{(bdc)}$ interactions at the *re*-face and π-stacking between the substrate's aromatic ring and that of the MOF's linker at the *si*-face.

2. Materials synthesis

2.1 General remarks

All reactions were carried out in anhydrous solvents. The Al-MIL-101-NH_2 was synthesized and activated according to previously reported procedures.[11] (*D*)-Boc-Pro-Gly-OH was purchased from Sigma-Aldrich. (*L*)-Boc-Pro-Gly-OH, was purchased from Bachem AG. All others reactants were commercially available (Sigma-Aldrich) and were used without further purification. Liquid-state NMR spectra were recorded on a Brüker 250 MHz spectrometer. Chemical shifts were reported in parts per million (ppm) referenced to the appropriate solvent peak. Prior to NMR analysis, MOF samples were dissolved in a HF-H_2O/dmso d6 solution. The powder X-Ray diffraction (PXRD) measurements were carried using a Brüker D8 advance diffractometer equipped with a Lynx-Eye detector (CuKα radiation, wavelengths λ= 0.154178nm). The PXRD studies were performed at room temperature. N_2 isotherms at 77K were performed using a BELSORP-mini apparatus (BELJapan). The samples were outgassed under vacuum (≈10^{-4} mbar) at room temperature for 12h before starting the measurements. The specific surface was determined by the BET method. ICP-OES analysis were done using an Activa from Jobin Yvon. TEM pictures were obtained on a Jeol 2010 LaB6 microscope operating at 200 kV. A dispersion of the catalyst crushed in ethanol was deposited on standard holey carbon-covered copper TEM grids.

2.2 Peptide coupling[10]

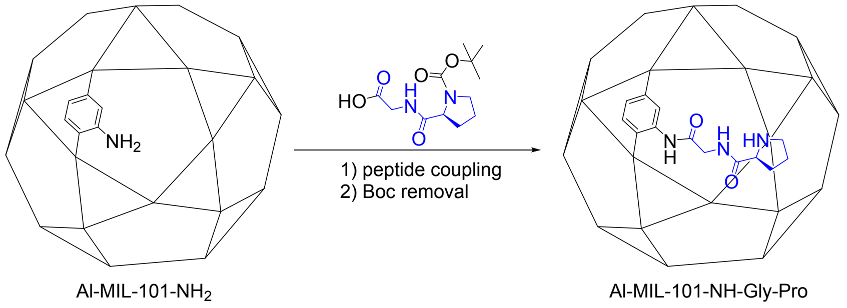

In a 10 mL microwave glass vial, 0.50 mmol of 2-Chloro-1-methyl-pyridinium iodide (Mukaiyama agent, 124 mg), 1.2 mmol of EtN^iPr_2 (DIEA, 112 µL) and 0.50 mmol of the Boc-Pro-Gly-OH (123 mg) either in its (*L*) or (*D*) form and the desired amount of Al-MIL-101-NH_2 (ca. 0.225 mmol -NH_2) were suspended in 5 mL of anhydrous dichloromethane. The resulting suspension was allowed to react under microwave irradiations for 20 minutes at 80°C (300 watts) under air cooling. The resulting suspension was centrifuged and the solid washed with dichloromethane (3 x 5 mL) to give a fine yellow powder after drying under vacuum at room temperature. The solid was finally characterized by powder X-ray diffraction, ^1H NMR and N_2 sorption analysis. Following this procedure, around 30 % of the amino groups were converted into the corresponding amide in Al-MIL-101-NH-Gly-Pro-Boc.

Then, in a 10 mL microwave glass vial, the desired Al-MIL-101-NH-Gly-Pro-Boc was suspended in 5 mL of anhydrous dichloromethane. The resulting suspension was allowed to

react under microwave irradiation for 10 minutes at 150°C (300 watts). The resulting suspension was centrifuged and the solid washed with dichloromethane (3 x 5 mL) to give the desired product Al-MIL-101-NH-Gly-Pro as a fine yellow powder after drying under vacuum at room temperature.

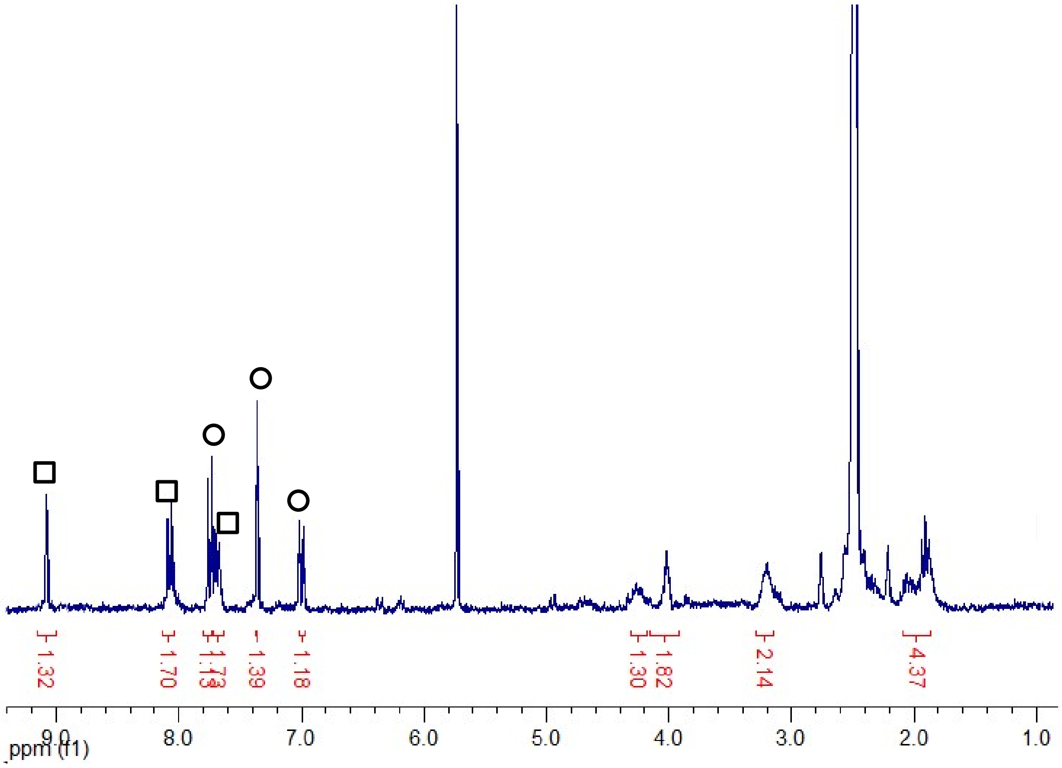

Figure S10. ^{1}H NMR spectrum of MIL-101-NH-Gly-(*L*)Pro dissolved in HF-H_2O/dmso d^6 solution; (ca. 30% modified, water signal suppression applied). Aromatic protons of unmodified and functionalized MOF linkers are indicated by circles and squares, respectively.

2.3 Ruthenium coordination

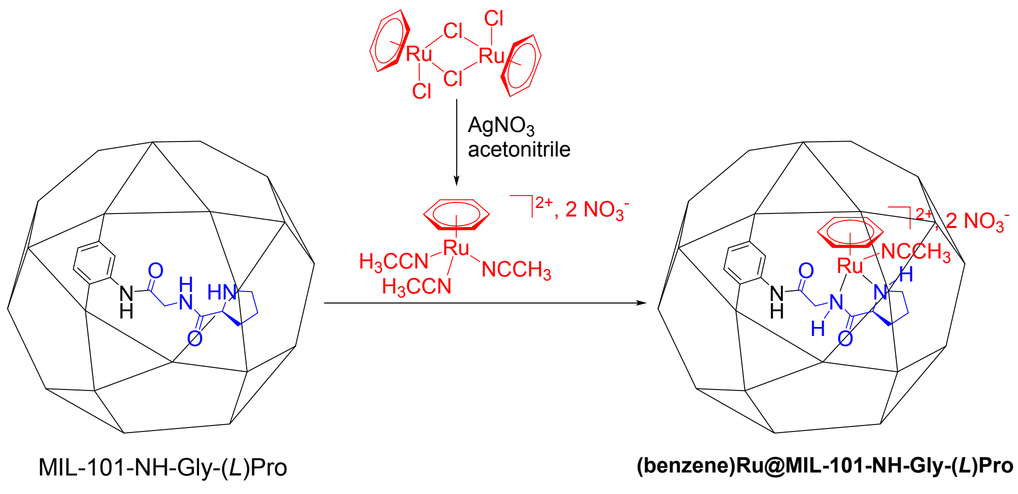

In a Schlenk tube, 56 mg of $[(C_6H_6)RuCl_2]_2$ (56 mg, 0.225 mmol Ru) were dissolved in 3 mL of dry acetonitrile and Ag_2NO_3 (0.225 mmol) were added. After stirring for two hours at 40°C, the mixture was filtered to remove the withe precipitate of silver chloride. Then, the obtained yellow solution was added to 35 mg of Al-MIL-101-NH-Gly-Pro (0.078 mmol GlyPro) either in its (*L*) or (*D*) form previously activated under vacuum at room temperature. The suspension was stirred at room temperature for 18 hours. Then, the solid was isolated by centrifugation, washed with acetonitrile (3 × 5mL), then isopropanol (3 × 5mL) and dichloromethane (3 × 5mL) and finally dried at room temperature under vacuum. The Al-MIL-101-NH-Gly-Pro$[Ru(C_6H_6)CH_3CN](NO_3)_2$ named **(benzene)Ru@MIL-101-NH-Gly-Pro** in the (*L*) or (*D*) form was obtained as a brownish solid.

3. Materials characterizations

3.1 Powder X-ray diffraction

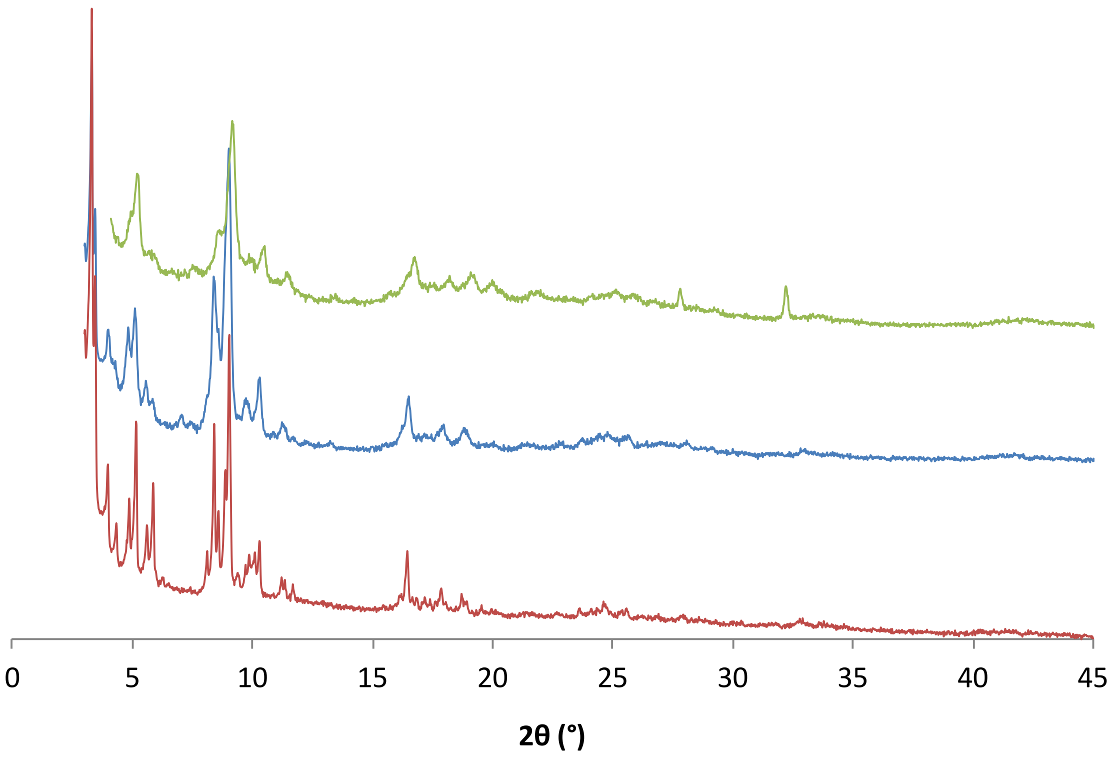

Figure S11. Power X-ray diffraction patterns of functionalized Al-MIL-NH$_2$. MIL-101-NH-Gly-(*L*)Pro (red), (benzene)Ru@MIL-101-NH-Gly-(*L*)Pro (blue), (benzene)Ru@MIL-101-NH-Gly-(*D*)Pro (green).

3.2 Nitrogen physisorption

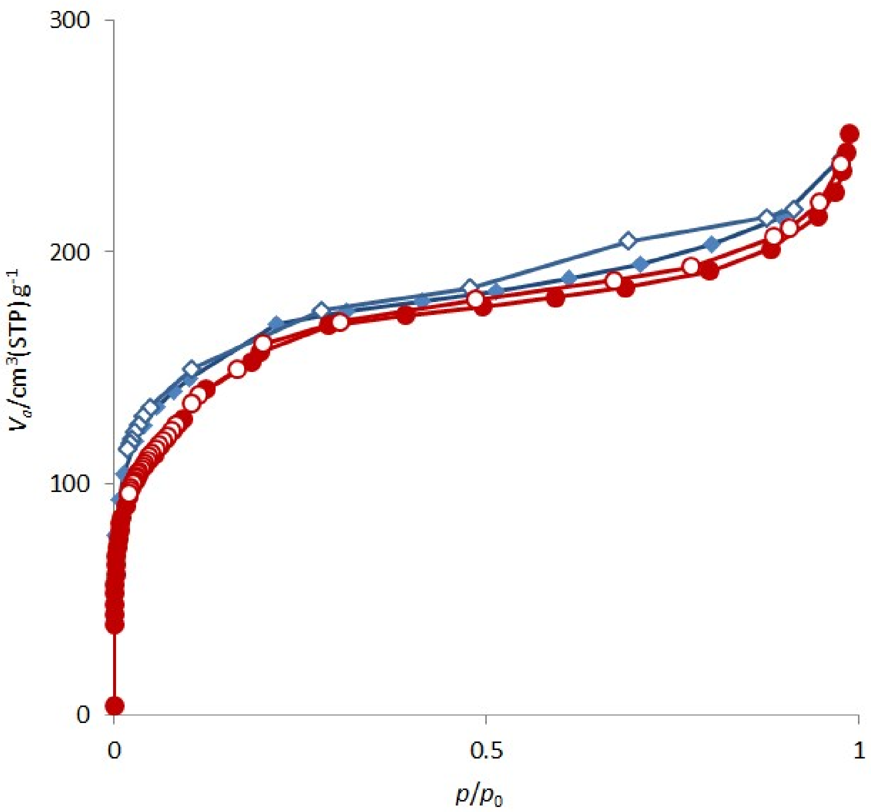

Figure S12. Nitrogen physisorption isotherms of the (benzene)Ru@MIL-101-NH-Gly-Pro solids. Adsorption and desorption are indicated by closed and open symbols, respectively, for (benzene)Ru@MIL-101-NH-Gly-(*L*)Pro (blue, diamonds) and (benzene)@MIL-101-NH-Gly-(*D*)Pro (red, circles).

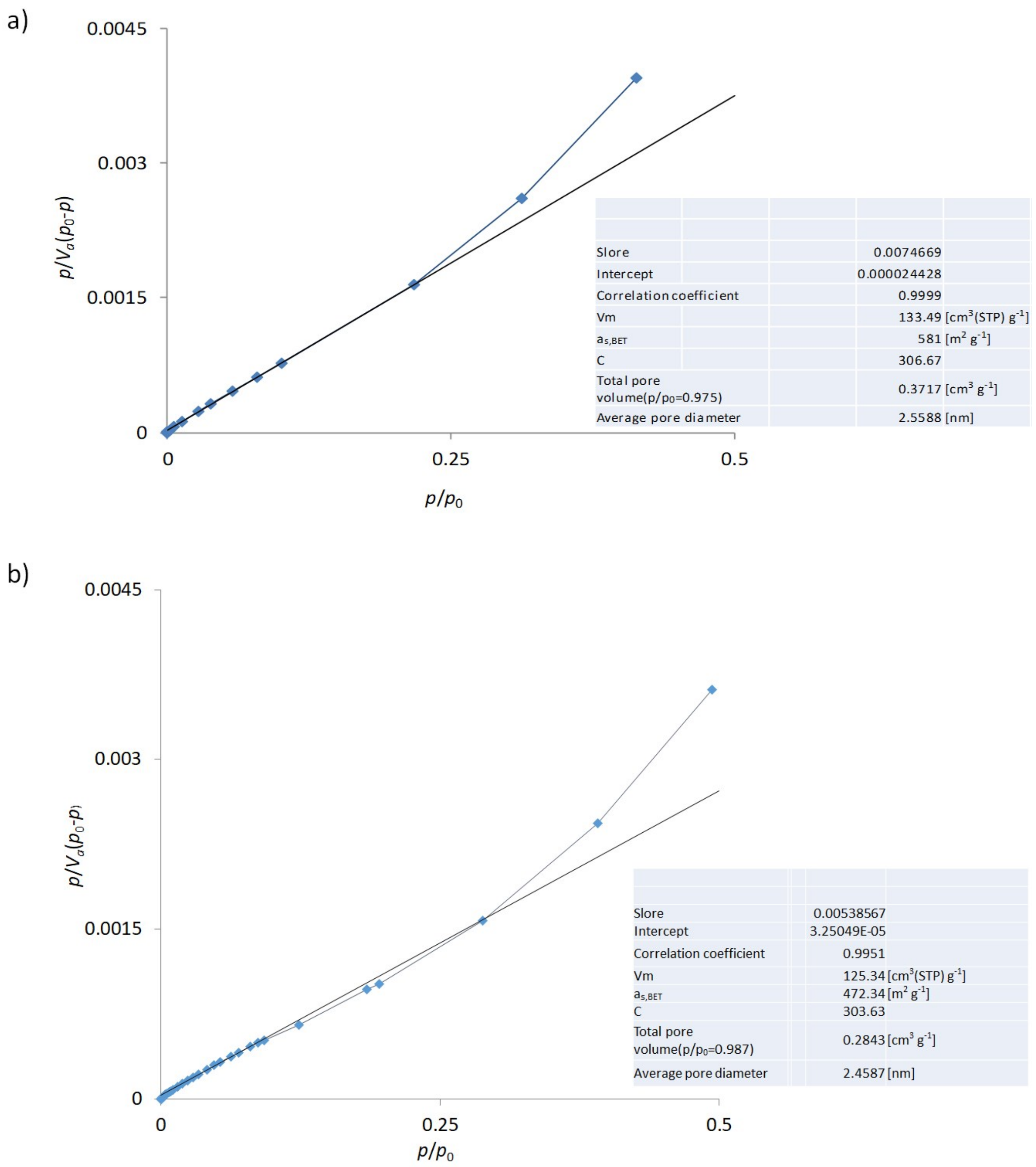

Figure S13. BET plot calculated from the N_2 physisorption isotherms of the (benzene)Ru@MIL-101-NH-Gly-Pro solids. (a) (benzene)Ru@MIL-101-NH-Gly-(*L*)Pro; (b) (benzene)Ru@MIL-101-NH-Gly-(*D*)Pro.

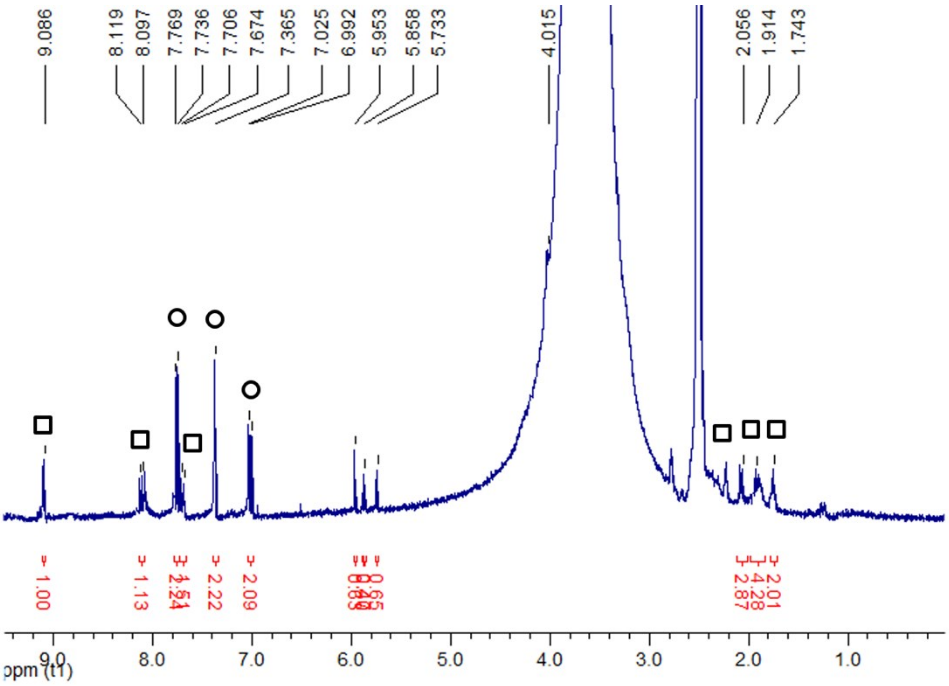

Figure S14. ^{1}H NMR spectrum of (benzene)Ru@MIL-101-NH-Gly-(*L*)Pro dissolved in HF-H_2O/dmso d^6 solution. Protons of unmodified and functionalized linkers are indicated by circles and squares, respectively.

3.3 Electron microscopy

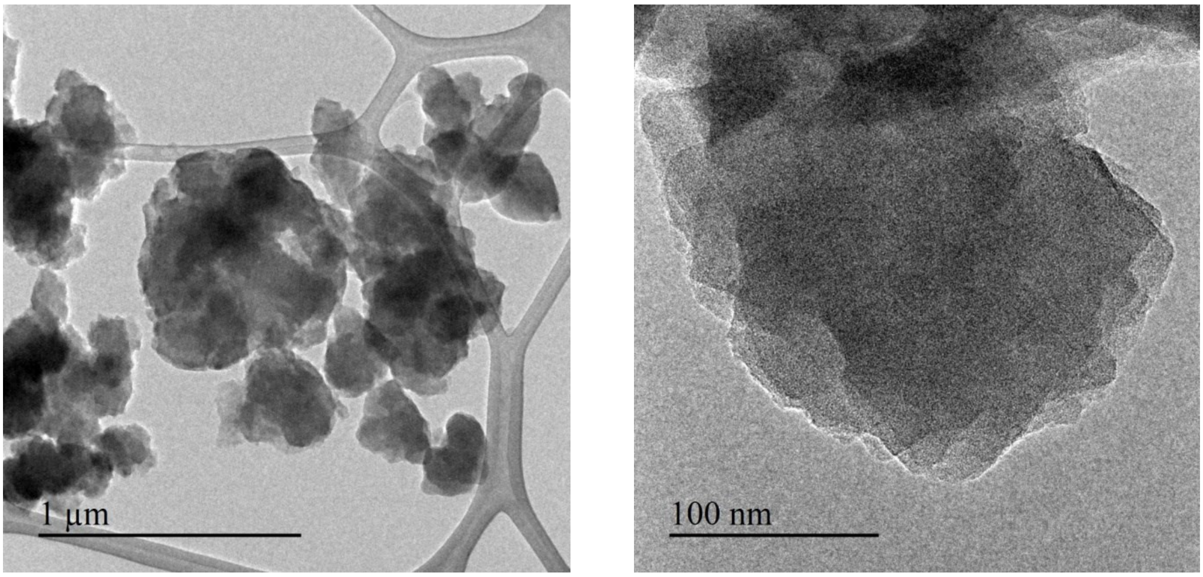

Figure S15. TEM micrographs of (benzene)Ru@MIL-101-NH-Gly-(*L*)Pro.

4. Synthesis of L^1 and L^2 molecular ligands

Synthesis of L^1

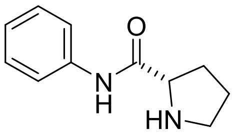

L^1

The (*S*)-*N*-phenylpyrrolidine-2-carboxamide **L^1** was prepared following the previously published procedure.[12] Boc-(*L*)Pro-Gly-OH (5 mmol, 1.07 g) was dissolved in dry THF (15 mL) with triethylamine (5 mmol, 0.5 g, 0.69 mL). The mixture was cooled to 0 °C and ethyl chloroformate (0.5 g, 5 mmol) was added dropwise. After stirring for 30 min at 0 °C, the aniline (5 mmol) was added. Then the mixture was stirred for 1 h at 0 °C and 16 h at room temperature. Finally the solvent was removed in vacuo and the crude residue was dissolved in ethyl acetate, washed with brine, and dried over $MgSO_4$. After solvent evaporation, the crude product was subjected to Boc-deprotection. A solution of Boc-protected Boc-L^1 (*ca.* 5 mmol) in dichloromethane (5 mL) was cooled to 0 °C. Then trifluoroacetic acid (5 mL) was added dropwise and the mixture stirred at room temperature for 2 h. Then solvent and unreacted trifluoroaecetic acid were removed *in vacuo*. The oily residue was diluted in dichloromethane (10 mL), washed with saturated K_2CO_3 solution and brine and dried over $MgSO_4$. The product **L^1** was obtained as a colorless oil in 60% yield (1.7g).
The NMR analysis data corresponds to those already reported.[13] ^{1}H NMR (400 MHz, $CDCl_3$, 25°C) δ ppm 8.45 (d, J = 2.70 Hz, 1H), 8.24- 8.35 (m, 1H), 8.17–8.22 (m, 1H), 7.18–7.28 (m, 2H), 3.85–3.90 (m, 1H), 2.94–3.17 (m, 2H), 1.95–2.23 (m, 2H), 1.71–1.84 (m, 2H).

Synthesis of L^2

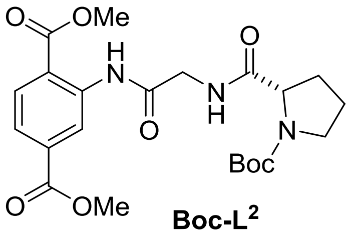

Boc-L^2

Boc-(*L*)Pro-Gly-OH (2.0 g, 7.34 mmol) and Et_3N (1.0 mL, 0.74 mmol) were dissolved in 25 mL of THF. The solution was cooled to 0 °C, dropwise of ethylchloroformate (0.7 mL, 0.80 mmol) was added for 15 min. After the solution was stirred for 30 min, dimethylaminoterephthalate (1.5 g, 7.34 mmol) dissolved in 9 mL of THF was added for 15 min. The resulting solution was stirred at 0 °C for 1 h, at room temperature for 16 h and then heated at reflux for 3 h. After cooling to room temperature, the solution was diluted with AcOEt (20 mL), filtered and then quenched with HCl aqueous solution (2*50 mL, 2.4 M). The organic layer was extracted with AcOEt (2*50 mL) and then washed with water (50 mL) and brine (50 mL).The organic layer was dried over Na_2SO_4, filtered and concentrated under reduced pressure. After purification by chromatography on silica gel using hexane/AcOEt : 3/7 as eluent, the compound **Boc-L^2** (1.7 g, 49% yield) was obtained.

^{1}H NMR (400 MHz, $CDCl_3$, 25°C) δ ppm 11.29 (br s, 1H), 9.28 (d, J = 1.0 Hz, 1H), 8.08 (d, J = 8.3 Hz, 1H), 7.75 (dd, J = 8.3 Hz, J = 1.4 Hz, 1H), 4.50-4.29 (m, 1H), 4.20-4.06 (m, 2H), 3.93 (s, 6H), 3.51-3.39 (m, 2H), 2.47-2.21 (m, 1H), 2.01-1.87 (m, 2H), 1.73-1.65 (m, 1H), 1.47 (s, 9H).

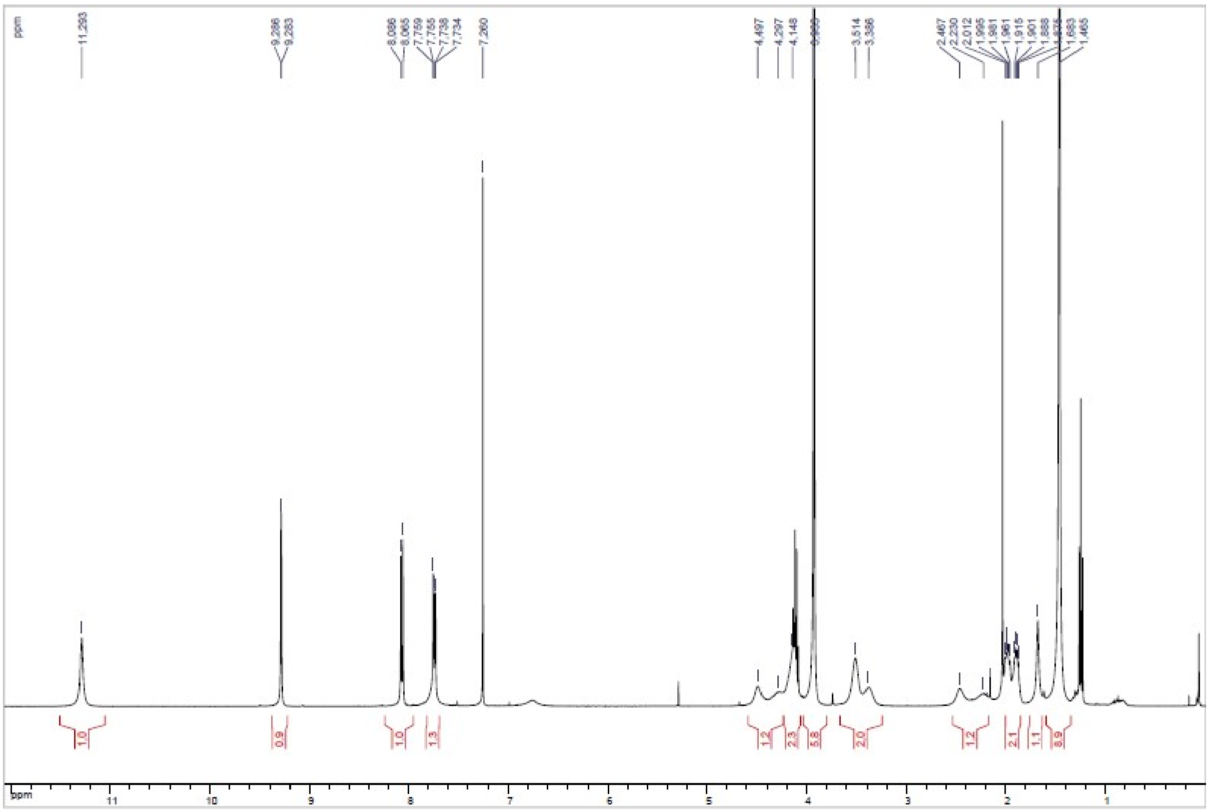

Figure S16. ^{1}H NMR spectrum of the molecular ligand Boc-L^2.

The compound **Boc-L^2** (150 mg, 0.32 mmol) was dissolved in DCM (3 mL). CF_3CO_2H (0.3 mL, 3.92 mmol) was added at room temperature. The resulting mixture was stirred overnight at room temperature and then evaporated. The residue was diluted with DCM (5 mL) and filtered through a pad of K_2CO_3. After concentration under reduced pressure, the compound **L^2** (104 mg, 90% yield) was obtained.

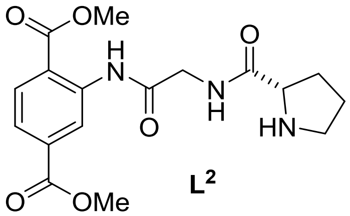

L^2

^{1}H NMR (400 MHz, $CDCl_3$, 25°C) δ ppm 11.33 (s, 1H), 9.32 (d, J = 1.4 Hz, 1H), 8.38-8.35 (m, 1H), 8.06 (d, J = 8.3 Hz, 1H), 7.73 (dd, J = 8.3 Hz, J = 1.6 Hz, 1H), 4.25 (dd, J = 17.3 Hz, J = 6.7 Hz, 1H), 4.05 (dd, J = 17.3 Hz, J = 5.6 Hz, 1H), 3.92 (d, J = 3.1 Hz, 6H), 3.92-3.89 (m, 1H), 3.09-2.99 (m, 2H), 2.25-2.16 (m, 2H), 2.05-1.97 (m, 1H), 1.87-1.68 (m, 2H). ^{13}C NMR (100 MHz, $CDCl_3$, 25°C) δ ppm 176.2, 168.3, 168.0, 166.0, 140.8, 135.4, 130.8, 123.6, 121.3, 118.4, 60.7, 52.6, 52.5, 47.3, 43.8, 30.4, 26.4. HRMS (ESI) m/z calcd for $C_{17}H_{22}N_3O_6$ [M+H]$^+$ 364.1503. Found 364.1490.

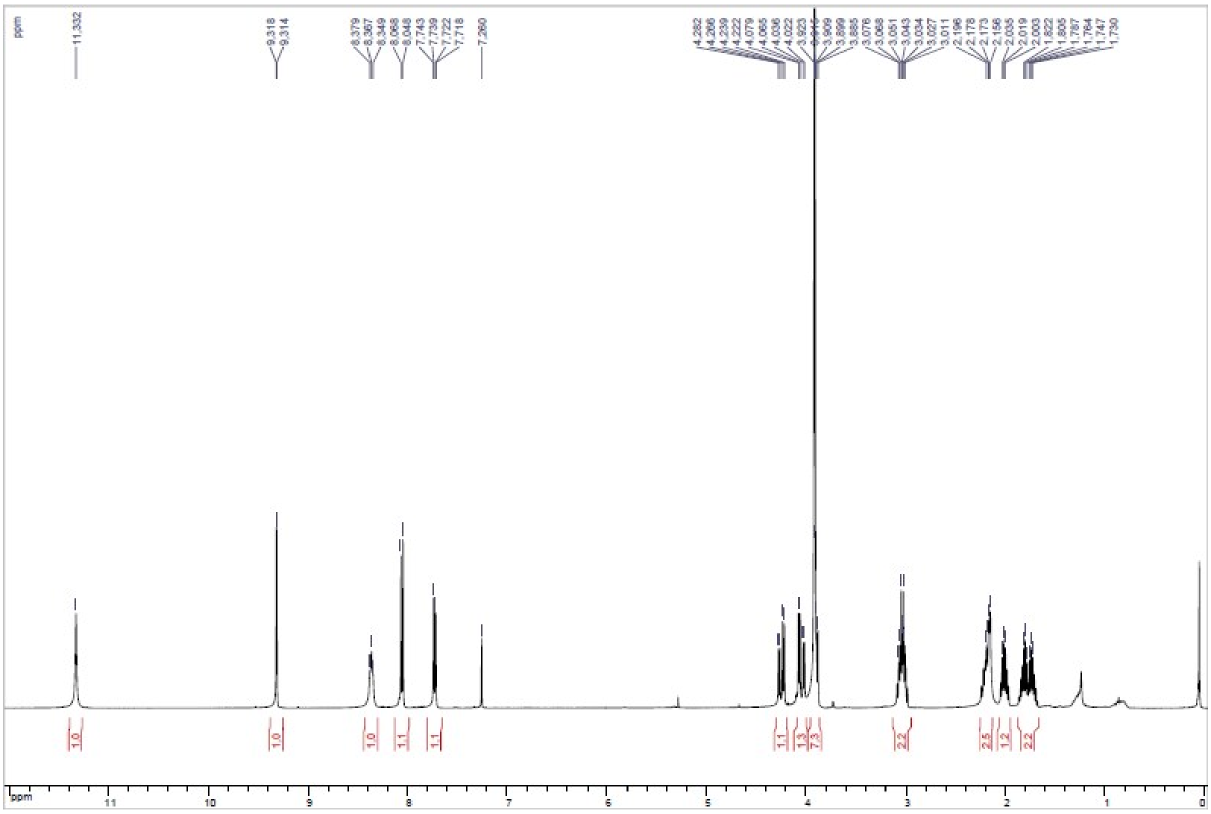

Figure S17. ^{1}H NMR spectrum of the molecular ligand L^2.

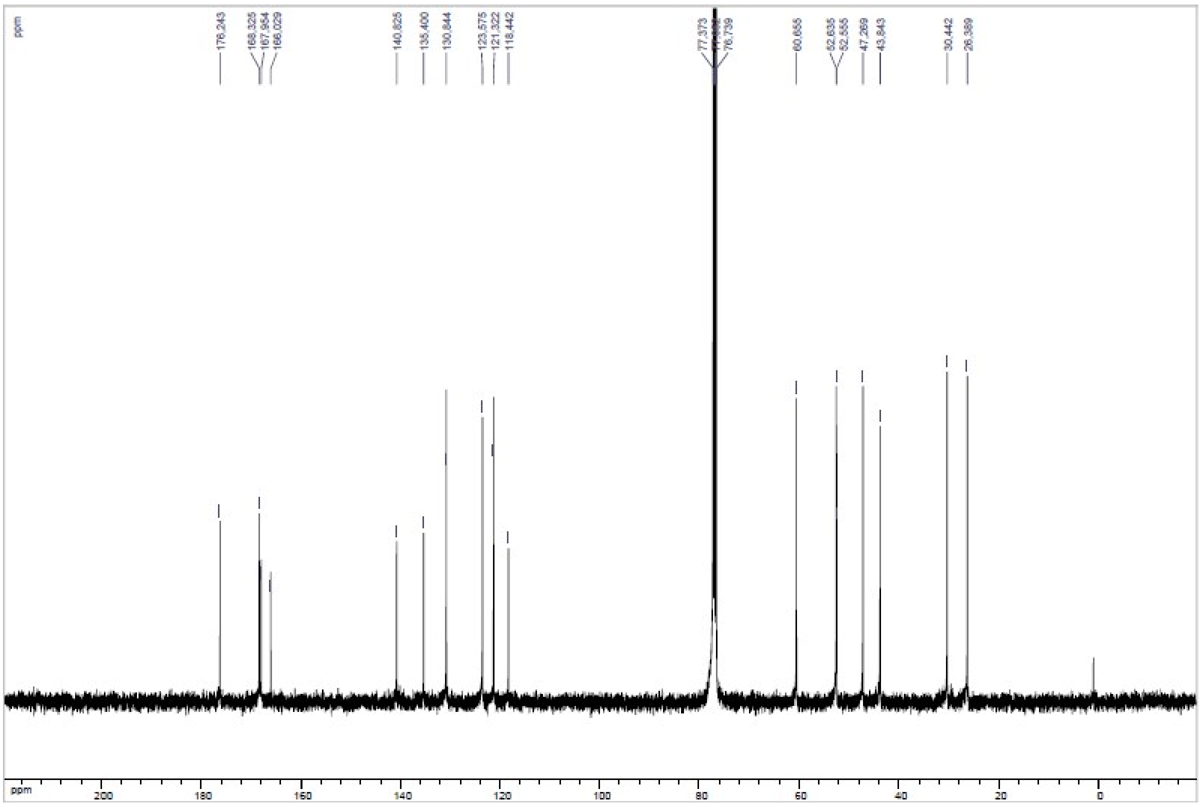

Figure S18. ^{13}C NMR spectrum of the molecular ligand L^2

5. Catalytic ketone transfer hydrogenation

5.1 Heterogeneous catalysis conditions

To (benzene)Ru@MIL-101-NH-Gly-Pro (10 mg, 3.9 µmol) was added a solution of KOH (2 mg, 36 µmol) in iPrOH (1 mL) and then heated at 80 °C for 4 h. To the resulting reddish suspension, acetophenone (45 µL, 386 µmol) was added and stirred for 18 h. The reaction mixture was then diluted with Et_2O (10 mL), filtered and washed with brine (10 mL). The organic layer was dried over $MgSO_4$ and filtered through a Millipore filter prior to analysis. The organic phases are combined, dried using magnesium sulphate and analyzed by HPLC (AS-H column, hexane:isopropanol = 97:3, 0.9 mL/min, 215 nm) to obtain conversion and enantiomeric excess (e.e.).

5.2 Homogeneous catalysis conditions

A suspension formed by mixing of $[C_6H_6RuCl_2]_2$ (8.5 mg, 0.017 mmol) and the ligand (0.033 mmol) in iPrOH (3 mL) was heated at 80 °C for 4 h. To the resulting orange solution, a solution of KOH (9.24 mg, 0.25 mmol) in iPrOH (1 mL) was added and stirred for 5 min at room temperature.Acetophenone (38 µL, 0.33 mmol) was added to the resulting red-brown solution. The reaction mixture was stirred at room temperature for 18 h and then diluted with Et_2O (10 mL) and washed with brine (10 mL). The organic layer was dried over $MgSO_4$ and filtered through a Millipore filter prior to analysis. The organic phases are combined, dried using magnesium sulphate and analyzed by HPLC (AS-H column, hexane:isopropanol = 97:3, 0.9 mL/min, 215 nm) to obtain conversion and enantiomeric excess (e.e.).

5.3 HPLC analysis

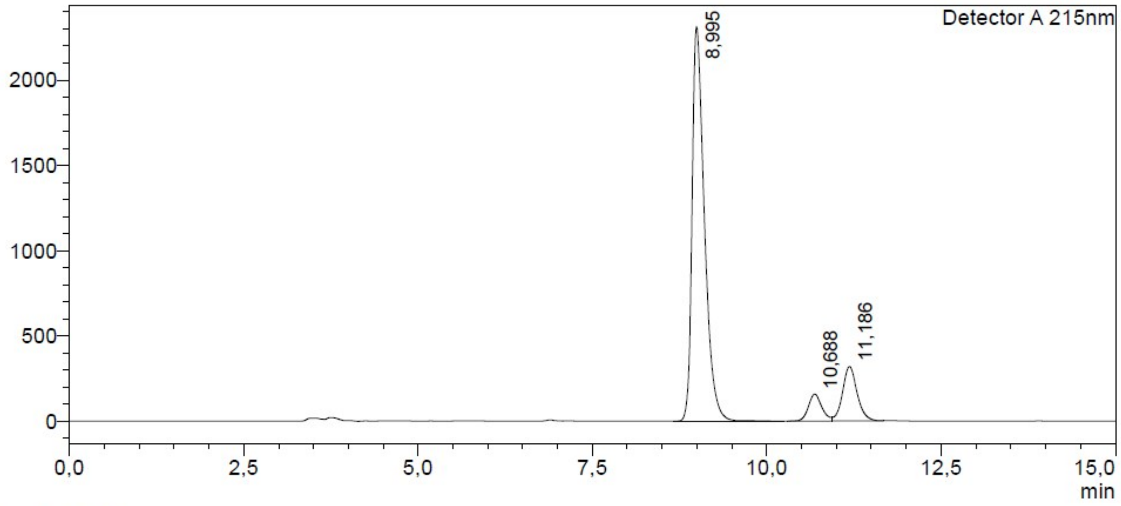

<Peak Table>

Detector A 215nm

Peak#	Ret. Time	Area	Height	Conc.	Unit	Mark	Name
1	8,995	28418481	2308539	0,000			acetophenone
2	10,688	2118085	158145	0,000			R-phenylethanol
3	11,186	4620231	318074	0,000		V	S-phenylethanol
Total		35156798	2784758				

Figure S19. Typical chromatogram and retention times obtained for acetophenone the ATH reaction catalyzed by **(benzene)Ru@MIL-101-NH-Gly-(*L*)Pro**.

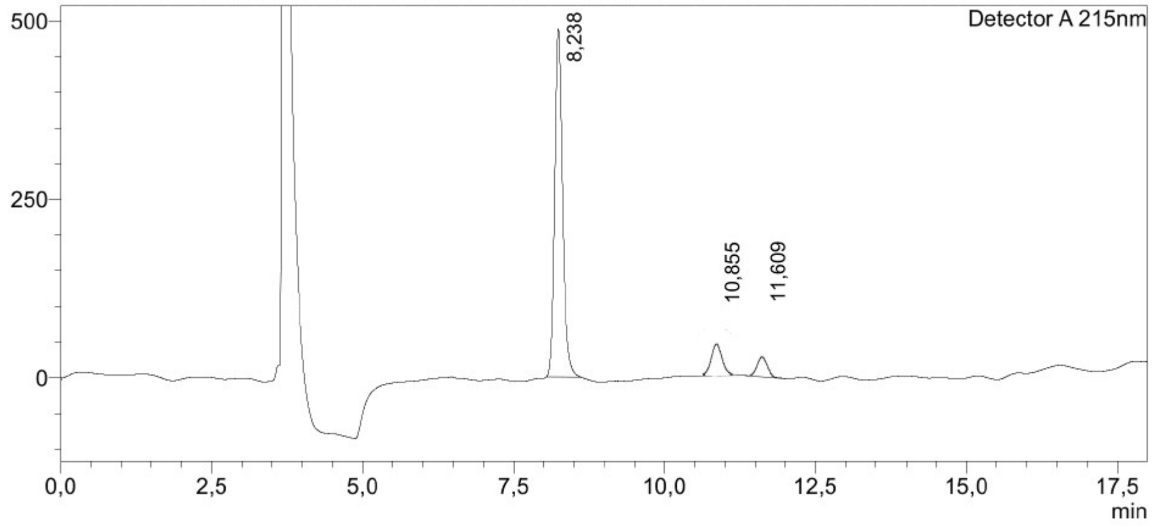

<Peak Table>

Detector A 215nm

Peak#	Ret. Time	Area	Height	Conc.	Unit	Mark	Name
1	8,238	4439500	486897	0,000		M	acetophenone
2	10,855	422826	65097	0,000		M	R-phenylethanol
3	11,609	281398	43898	0,000		M	S-phenylethanol
Total		5143724	595892				

Figure S20. Typical chromatogram and retention times obtained for the acetophenone ATH reaction catalyzed by **(benzene)Ru@MIL-101-NH-Gly-(*D*)Pro**.

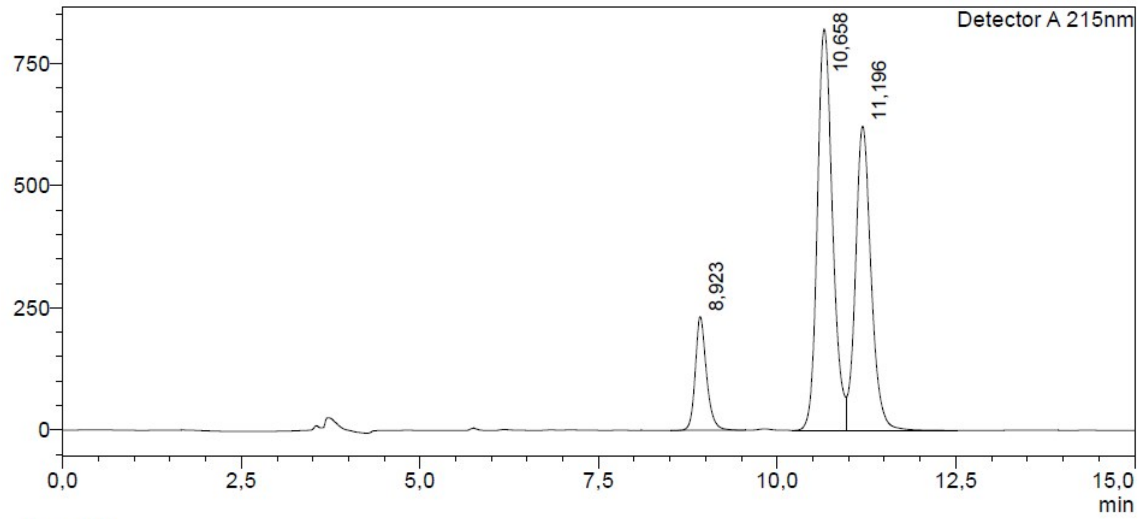

<Peak Table>

Detector A 215nm

Peak#	Ret. Time	Area	Height	Conc.	Unit	Mark	Name
1	8,923	2547183	232597	0,000			acetophenone
2	10,658	12150291	820055	0,000			R-phenylethanol
3	11,196	9482476	621809	0,000		V	S-phenylethanol
Total		24179950	1674461				

Figure S21. Typical chromatogram and retention times obtained for the acetophenone ATH reaction catalyzed by $(C_6H_6)Ru(\mathbf{L^1})Cl_2$.

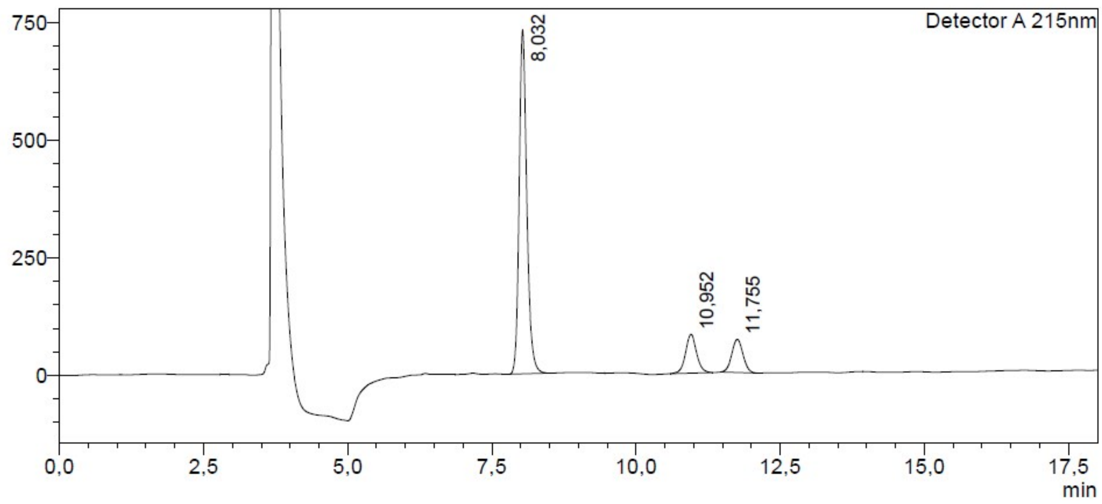

<Peak Table>

Detector A 215nm

Peak#	Ret. Time	Area	Height	Conc.	Unit	Mark	Name
1	8,032	6717733	729711	0,000		M	acetophenone
2	10,952	1094602	82325	0,000		M	R-phenylethanol
3	11,755	951130	70557	0,000		M	S-phenylethanol
Total		8763465	882593				

Figure S22. Typical chromatogram and retention times obtained for the acetophenone ATH reaction catalyzed by $(C_6H_6)Ru(\mathbf{L^2})Cl_2$.

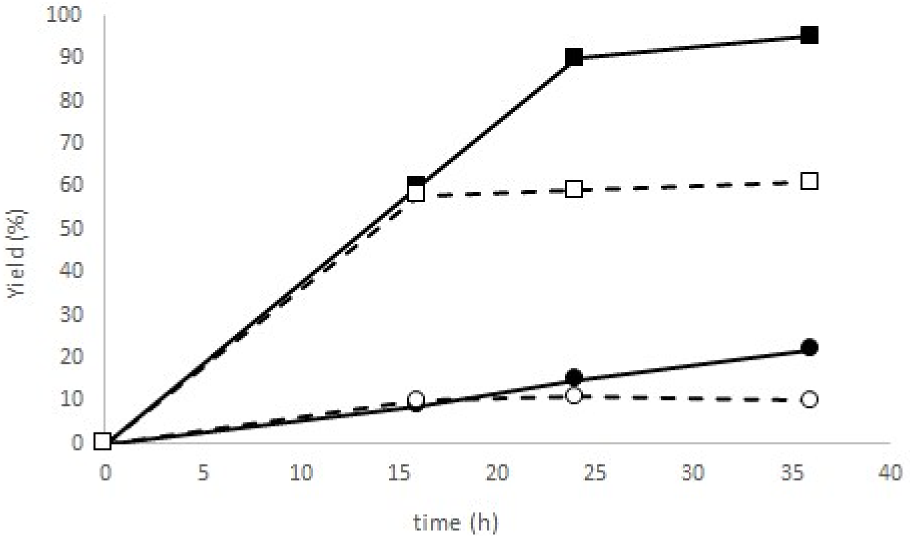

Figure S23. Acetophenone ATH reaction catalyzed by (benzene)Ru@MIL-101-NH-Gly-(*L*)Pro. Experiments were performed at 20°C (●) and 60°c (■). The dashed lines display the evolution of the yield inside the solution after catalyst removal by filtration at 20°C (○) and 60°C (□).

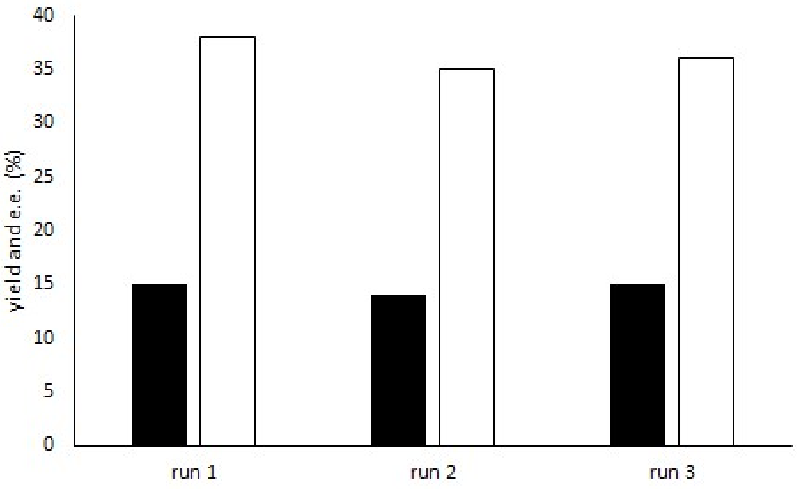

Figure S24. **Recycling experiments for acetophenone ATH reaction catalyzed by (benzene)Ru@MIL-101-NH-Gly-(*L*)Pro**. Experiments were performed at 20°C for 24 hours. Yields are shown in black and e.e. in white.

5.4 DFT computations of the {(benzene)Ru(L²), acetophenone} molecular complex

The molecular complexes and **(benzene)Ru(L²)** were optimized at the B3LYP-D3[14,15,16,7]/def 2-TZVP[17] level of density functional theory using the Gaussian 09 software package.[18] A quasi-relativistic Stuttgart-Dresden effective core potential was used on Ru.[19] Frequency calculations on optimized geometries ensured that structures were minima (zero imaginary frequency) on the potential energy surface.

Our calculations show that with the **(benzene)Ru(L²)** molecular catalyst, the *si*-face of the acetophenone substrate is favored when compared to its *re*-face, with a difference in interaction energies, $\delta\Delta E_{inter}$(*re-si*), of +10.6 kJ mol^{-1}, which in is line with the enantioselectivity observed in favor of the *R*-alcohol product. The *si*-face allows a slightly shorter $C_{(C=O)}\cdots H_{Ru}$ interaction at 2.6 Å than at the *re*-face ($C_{(C=O)}\cdots H_{Ru}$ =2.7 Å), besides the CH/π lateral stabilization with one hydrogen atom of the Glycine –CH_2 group.

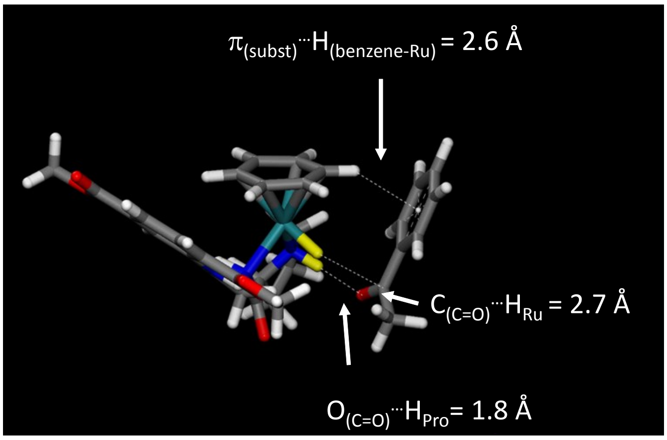

Figure S25. *Re*-face approach towards (C_6H_6)Ru(L²) molecular catalyst. At the *re*-face of acetophenone, the $O_{(C=O)}\ldots H_{Pro}$ hydrogen bond occurs at 1.8 Å and the $C_{(C=O)}\cdots H_{Ru}$ interaction at 2.8 Å. The aromatic ring of the substrate is stabilized by a $\pi_{(subs)}\cdots H_{(benzene\text{-}Ru)}$ interactions at 2.6 Å (distance to the centroid of the aromatic ring).

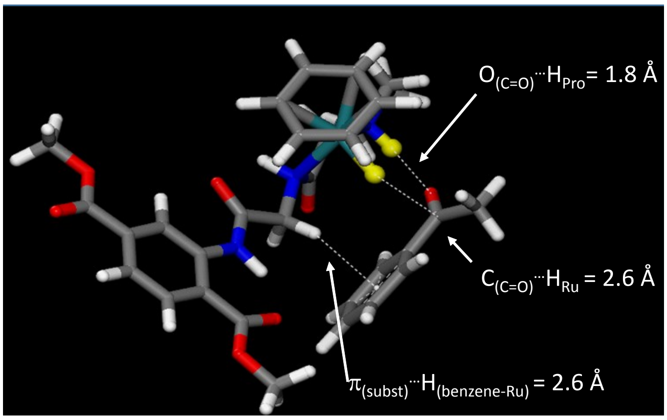

Figure S26. *Si*-face approach towards (C_6H_6)Ru(L²) molecular catalyst. At the *si*-face, the $O_{(C=O)}\ldots H_{Pro}$ hydrogen bond occurs at 1.8 Å and the $C_{(C=O)}\ldots H_{Ru}$ interaction at 2.6 Å, at a shorter distance in comparison with the *re*-face. The occurrence of CH/π interactions with the glycine –CH_2 group at the *si*-face of the substrate stabilizes the aromatic ring of the substrate.

Table S1. Structural a[illegible]
(benzene)Ru@MIL-101-N[illegible]
exposing acetophenone throu[illegible]
1, 2, **3** and **4**. The difference[illegible]
computed at the DFT-D3 lev[illegible]
product.

Variant	Config°	Host-guest interactions at the *re*-face (Å)			Host-guest interactions at the *si*-face (Å)			$\delta\Delta E_{inter}$ (*re-si*) (kJ.mol[-1])	Product
		$O_{(C=O)}\cdots H_{Pro}$	$C_{(C=O)}\cdots H_{Ru}$	Lateral stabilization	$O_{(C=O)}\cdots H_{Pro}$	$C_{(C=O)}\cdots H_{Ru}$	Lateral stabilization		
1	*S-S-R-S*	1.[illegible]	2.6	$\pi_{(substrate)}\cdots H_2N_{(bdc)}$ 2.6*	1.8	2.7	$\pi_{(substrate)}\cdots H_{(benzene-Ru)}$ 2.9**	-15.7	S >> R
2	*S-S-S-S*	1.[illegible]	2.6	$CH_{(substrate)}\cdots \pi_{(bdc)}$ 2.6*	1.8	2.9	$\pi_{(substrate)}\cdots H_{(benzene-Ru)}$ 3.2**<br>$CH_{(substrate)}\cdots \pi_{(bdc)}$ 2.9**	-3.0	S>R
3	*R-S-R-R*	1.[illegible]	2.7	$CH_{3(substrate)}\cdots \pi_{(bdc)}$ 3.2*<br>$CH_{3(substrate)}\cdots NH_{2(bdc)}$ 2.9<br>$\pi_{(substrate)}\cdots CH_{(Pro)}$ 2.9**	1.8	3.2	$CH_{(substrate)}\cdots \pi_{(bdc)}$ 2.9*	-18.1	S >>R
4	*R-S-S-R*	1.[illegible]	2.6	$CH_{3(substrate)}\cdots NH_{2(bdc)}$ 2.7<br>$CH_{3(substrate)}\cdots \pi_{(bdc)}$ 3.4*<br>$\pi_{(substrate)}\cdots CH_{(Pro)}$ 2.9**	1.9 (1.9)[a]	2.7	$\pi_{(substrate)}\cdots \pi_{(bdc)}$ stacking 3.6-3.8***	-1.6	S~ R
L[2]	*R-S-R-R*	1.[illegible]	2.8	$CH_{(Cp)}$-$\pi_{(substrate)}$ 2.6 **	1.8	2.6	$CH_{(Gly)}$-$\pi_{(substrate)}$ 2.6 **	+10.6	R>S

(*) = distance to centroid of the (bdc)$_{MOF}$ aromatic ring; (**) = distance to the centroid of the acetophenone's aromatic ring. (***)= distance between an aromatic carbon of the substrate and the centroid of the (bdc)$_{MOF}$ aromatic ring. (a): additional interaction with the glycine residue, through an $\mathbf{O}_{(C=O)}\cdots\mathbf{H}_{Gly}$ interaction.

Within the first coordination sphere, the hydrogenation of acetophenone into alcohol requires the adequate positioning of its C=O group with respect to the hydridic H_{Ru} and vicinal protic $H_{N(Pro)}$ hydrogen atom of the proline, in order to form the $C_{(C=O)}\cdots H_{Ru}$ and $O\cdots H_{N(Pro)}$ interactions required for the subsequent ATH reaction. At the *re*-face of acetophenone, a close approach of the C=O group towards the hydridic H_{Ru} and protic H_{Pro} hydrogens is systematically found in all variants 1-4, whereby $O_{(C=O)}\cdots H_{Pro}$ hydrogen bonds occur at 1.8-1.9 Å and $C_{(C=O)}\cdots H_{Ru}$ interactions at 2.6-2.7 Å. At the *si*-face of acetophenone, similar short $O_{(C=O)}\cdots H_{Pro}$ distances occur. However, they are assorted with a slightly larger range of $C_{(C=O)}\cdots H_{Ru}$ distances (2.7-3.2 Å), revealing weaker $C_{(C=O)}\cdots H_{Ru}$ interactions than those taking place in the *re*-face complexes. In addition, lateral stabilization emanating from host-guest interactions are detailed and highlight the role of the framework.

6. References

[1] (a) Férey, G.; Mellot-Draznieks, C.; Serre, C.; Millange, F.; Dutour, J.; Surblé, S.; Margiolaki, I. A chromium terephthalate-based solid with unusually large pore volumes and surface area. *Science* **2005**, *309*, 2040-2042. (b) Mellot-Draznieks C. and Férey, G. Assembling molecular species into 3D frameworks: Computational design and structure solution of hybrid materials*Prog. Solid State Chem.* **2005**, *33*, 187.

[2] Todorova, T. K.; Rosenska, X.; Gervais, C.; Legrand, A.; Ho, L. N.; Berruyer, P.; Lesage, A.,; Emsley, L.; Farrusseng, D.; Canivet, J.; Mellot-Draznieks C. Molecular Level Characterization of the Structure and Interactions in Peptide-Functionalized Metal-Organic Frameworks.*Chem.- Eur. J.* **2016**, *22*, 16531-16538.

[3] Hartmann, M., Fischer, M. Amino-functionalized basic catalysts with MIL-101 structure. *Microporous Mesoporous Mater.* **2012**, *164*, 38-43.

[4] (a) Kresse, G.; Furthmüller, J. Efficiency of ab-initio total energy calculations for metals and semiconductors using a plane-wave basis set. *Comput. Mater. Sci.* **1996**, *6*, 15-50. (b) Kresse, G.; Furthmüller, J. Efficient iterative schemes for ab initio total-energy calculations using a plane-wave basis set. *Phys. Rev. B* **1996**, *54*, 11169-11186.

[5] Rappé, A. K.; Casewit, C. J.; Colwell, K. S.; Goddard III, W. A.; Skiff, W. M. UFF, a Full Periodic Table Force Field for Molecular Mechanics and Molecular Dynamics Simulations. *J. Am. Chem. Soc.* **1992**, *114*, 10024.

[6] Perdew, J. P., Burke, K., Ernzerhof, M. Generalized gradient approximation made simple. *Phys. Rev. Lett.* **1996**, *77*, 3865-3868.

[7] Grimme, S.; Antony, J.; Ehrlich, S.; Krieg, H. A consistent and accurate ab initio parametrization of density functional dispersion correction (DFT-D) for the 94 elements H-Pu. *J. Chem. Phys.* **2010**, *132*, 154104.

[8] Blöchl, P. E. Projector Augmented-Wave Method. *Phys. Rev. B* **1994**, *50*, 17953-17979.

[9] Kresse, G., Joubert, D. *Phys. Rev. B* **1999**, *59*, 1758-1775.

[10] Noyori, R. & Hashiguchi, S. Asymmetric transfer hydrogenation catalyzed by chiral ruthenium complexes. *Acc. Chem. Res.* **1997**, *30*, 97-102.

[11] Bonnefoy, J.; Legrand, A.; Quadrelli, E. A.; Canivet, J. & Farrusseng, D. Enantiopure Peptide-Functionalized Metal–Organic Frameworks. *J. Am. Chem. Soc.* **2015**, 9409-9416.

[12] Kinsella, M.; Duggan, P. G. & Lennon, C. M. Screening of simple N-aryl and N-heteroaryl pyrrolidine amide organocatalysts for the enantioselective aldol reaction of acetone with isatin *Tetrahedron: Asymmetry* **2011**, *22*, 1423-1433.

[13] (a) Tang, Z.; Cun, L.-F.; Cui, X.; Mi, A.-Q.; Jiang, Y.-Z.; Gong, L.-Z. Design of highly enantioselective organocatalysts based on molecular recognition. *Org. Lett.* **2006**, *8*, 1263-1266. (b) Luo, S.; Xu, H.; Li, J.; Zhang, L.; Mi, X.; Zheng, X.; Cheng, J. P. Facile evolution of asymmetric organocatalysts in water assisted by surfactant Bronsted acids. *Tetrahedron* **2007**, *63*, 11307-11314.

[14] Becke, A. D. Density-Functional Thermochemistry. 3. The Role of the Exact Exchange. *J. Chem. Phys.* **1993**, *98*, 5648-5652.

[15] Stephens, P. J.; Devlin, F. J.; Chabalowski, C. F.; Frisch, M. J. *J. Phys. Chem.* **1994**, 98, 11623-11627.

[16] Grimme, S.; Ehrlich, S. & Goerigk, L. Effect of the Damping Function in Dispersion Corrected Density Functional Theory. *J. Comput. Chem.* **2011**, *32*, 1456-1465.

[17] Weigend F. & Ahlrichs, R. Balanced basis sets of split valence, triple zeta valence and quadruple zeta valence quality for H to Rn: Design and assessment of accuracy. *Phys.*

Chem. Chem. Phys. 2005, *7*, 3297-3305.

18 Frisch, M. J.; Trucks, G. W.; Schlegel, H. B.; Scuseria, G. E.; Robb, M. A.; Cheeseman, J. R.; Scalmani, G.; Barone, V.; Mennucci, B.; Petersson, G. A. et al., Gaussian 09; Gaussian, Inc.: Wallington CT., 2013.

19 Andrae, D.; Häußermann, U.; Dolg, M.; Stoll, H. & Preuß, H. Energy-Adjusted Ab Initio Potential for the 2nd and 3rd Row Transition-Elements. *Theor. Chim. Acta* **1990**, *77*, 123-141.
